# Molecular Structure and Phylogenetic Analyses of the Complete Chloroplast Genomes of Three Medicinal Plants *Conioselinum vaginatum*, *Ligusticum sinense*, and *Ligusticum jeholense*

**DOI:** 10.3389/fpls.2022.878263

**Published:** 2022-06-06

**Authors:** Xue-Ping Wei, Xiao-Yi Zhang, Yu-Qing Dong, Ji-Long Cheng, Yun-Jun Bai, Jiu-Shi Liu, Yao-Dong Qi, Ben-Gang Zhang, Hai-Tao Liu

**Affiliations:** ^1^Key Laboratory of Bioactive Substances and Resources Utilization of Chinese Herbal Medicine, Ministry of Education, Institute of Medicinal Plant Development, Chinese Academy of Medical Sciences, Peking Union Medical College, Beijing, China; ^2^Engineering Research Center of Tradition Chinese Medicine Resource, Ministry of Education, Institute of Medicinal Plant Development, Chinese Academy of Medical Sciences, Peking Union Medical College, Beijing, China; ^3^National Resource Center for Chinese Materia Medica, China Academy of Chinese Medical Sciences, Beijing, China

**Keywords:** Apiaceae, chloroplast genome, *Conioselinum vaginatum*, DNA barcode, *Ligusticum jeholense*, *Ligusticum sinense*, phylogeny

## Abstract

Most plants of *Ligusticum* have an important medicinal and economic value with a long history, *Ligusticum sinense* and *L. jeholense* (“Gaoben”) has long been used in traditional Chinese medicine for the treatment of carminative, dispelling cold, dehumidification, and analgesia. While in the market *Conioselinum vaginatum* (Xinjiang Gaoben) is substitution for Gaoben, and occupies a higher market share. These three Gaoben-related medicinal materials are similar in morphology, and are difficult to distinguish from each other by the commonly used DNA barcodes. The chloroplast genome has been widely used for molecular markers, evolutionary biology, and barcoding identification. In this study, the complete chloroplast genome sequences of *C. vaginatum*, *L. sinense*, and *L. jeholense* were reported. The results showed that the complete chloroplast genomes of these three species have typical quadripartite structures, which were comprised of 148,664, 148,539, and 148,497 bp. A total of 114 genes were identified, including 81 protein-coding genes (PCGs), 29 tRNA genes, and four rRNA genes. Our study indicated that highly variable region *ycf*2-*trn*L and *acc*D-*ycf*4 that can be used as specific DNA barcodes to distinguish and identify *C. vaginatum*, *L. sinense*, and *L. jeholense*. In addition, phylogenetic study showed that *C. vaginatum* nested in *Ligusticum* and as a sister group of *L. sinense* and *L. jeholense*, which suggested these two genera are both in need of revision. This study offer valuable information for future research in the identification of Gaoben-related medicinal materials and will benefit for further phylogenetic study of Apiaceae.

## Introduction

Apiaceae is one of the more evolved groups of angiosperms. There are about 250–440 (−455) genera and 3,300–3,700 species in the world, which are widely distributed in temperate regions (Sheh et al., 2005). The highly consistent umbel and two carpels are the most prominent characteristics of Apiaceae, which also makes Apiaceae the first recognized family and easy to identify among angiosperms. However, the high similarity of flower characteristics and dependence on fruit characteristics make it very difficult to identify at genus and species level, and Apiaceae has become a taxonomic difficult group (Constance, 1971).

Plants of Apiaceae play a certain role in the national economy. Many of them can be used as medicinal materials, vegetables, spices, pesticides, and so on. In terms of medicine, such as famous Chinese medicinal materials as *Angelica sinensis* (Oliv.) Diels, *Ligusticum sinense* Oliv., *Angelica dahurica* (Fisch. ex Hoffm.) Benth. et Hook. f. ex Franch. E, *Bupleurum chinense* DC., etc., these species enjoy a high reputation in the domestic and foreign markets. *Ligusticum* L. is a widespread, complex genus and the classification of which is controversial. The phylogenetic tree constructed with ITS and chloroplast genes also shows that *Ligusticum* is polyphyletic, and relationships with nearby genera such as *Conioselinum* Fisch. ex Hoffm were unclear (Downie et al., 2020).

**Figure 1:**
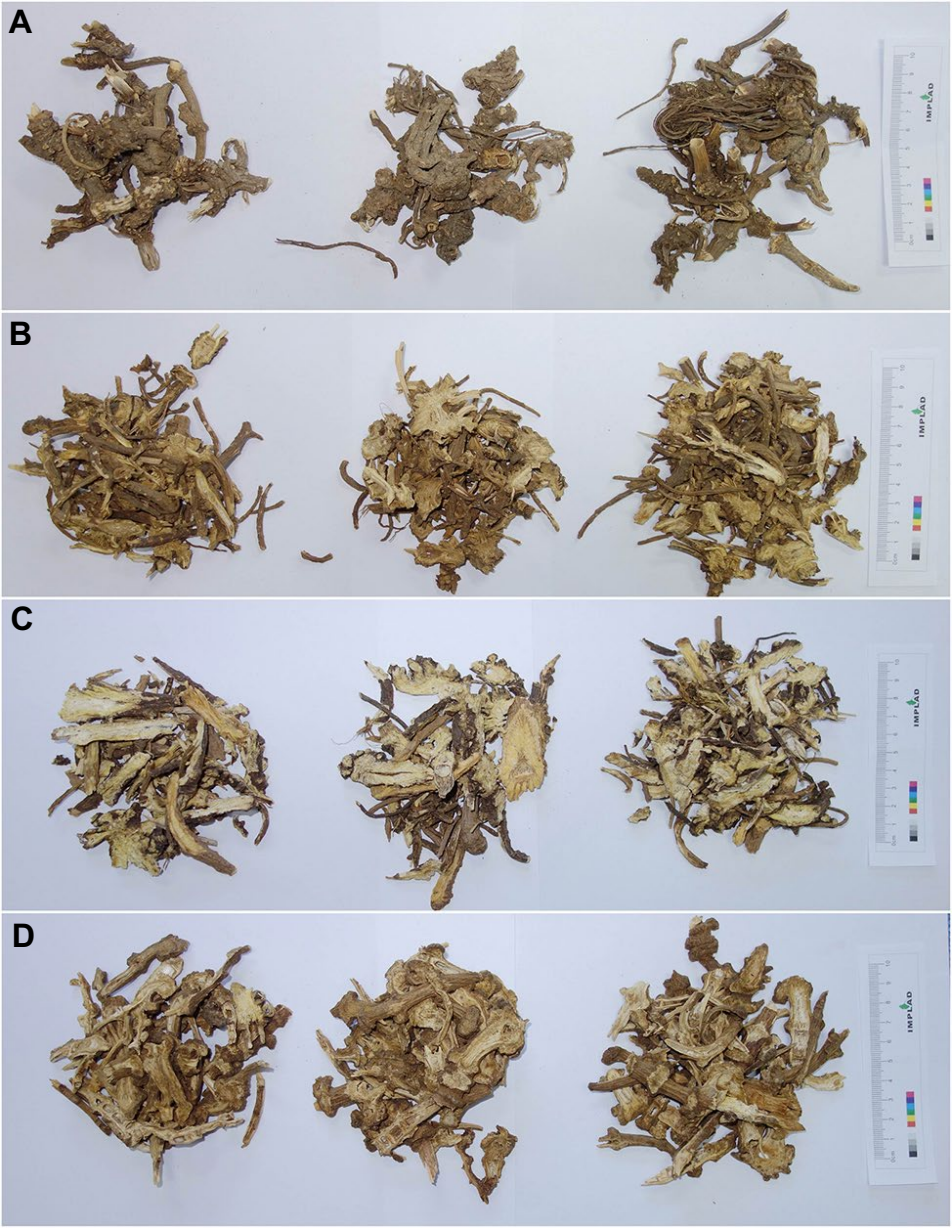
Gaoben-related medicinal materials in the market **(A)** *Ligusticum sinense*, **(B)** *Ligusticum jeholense*, **(C)** *Conioselinum vaginatum*, and **(D)** *Ligusticum* sp.

The traditional Chinese medicine Ligustici Rhizoma et Radix (“Gaoben” in Chinese) is the dried rhizome and root of *Ligusticum sinense* Oliv. (“Gaoben” in Chinese) and *Ligusticum jeholense* Nakai et Kitag (“Liao-Gaoben” in Chinese), recorded in the Chinese Pharmacopoeia (Chinese Pharmacopeia Commission, 2020; Figures 1A,B). Medicinal materials Gaoben (in the following, Gaoben represents Ligustici Rhizoma et Radix) has good traditional efficacy for dispelling wind, removing dampness, and relieving pain with a long history (Chinese Pharmacopeia Commission, 2020). Modern pharmacological studies have shown that Gaoben has potential neuroprotective and vasodilation activity and inhibits vascular smooth muscle cell proliferation and anti-inflammatory, analgesic, and antithrombotic activity (Zhang et al., 2001; Lu et al., 2006; Chan et al., 2007; Peng et al., 2007; Lin et al., 2011). Wild resources of Gaoben are gradually decreasing and its yield decreases sharply due to years of excessive collection and destruction of the ecological environment. According to a survey of the medicinal material market, Gaoben occupies a lower share, while Xinjiang Gaoben occupies nearly 70% of the market and is usually used as Gaoben (Zhao, 2007; Figure 1C). In addition, a small amount of other *Ligusticum* plant was traded as Gaoben (Figure 1D). Xinjiang Gaoben is the dried rhizome and root of the *Conioselinum vaginatum* (Spreng.) Thell., and is similar in appearance to Gaoben. Such substitutes entered the local medicinal materials market in the 1960 and 1970s and are commonly used as folk medicines in dispelling cold, carminative, and analgesia (Xinjiang Institute of biological soil, Chinese Academy of Sciences, 1981; Pan and Watson, 2005). Source plants of these three Gaoben-related medicinal materials are distributed in different places in China. Wild *L. sinense* are mainly distributed in Hubei, Sichuan, Shaanxi, Henan, Hunan, Jiangxi, and Zhejiang provinces, and is cultivated in many other provinces and regions. *L. jeholense* is found mainly in Hebei, Jilin, Liaoning, Shandong, and Shanxi provinces (Chen et al., 1985). *Conioselinum vaginatum* are grown mainly in mountain meadows, hillside grasses, and river valley shrubs and are distributed in the Tianshan Mountains, Altai Mountains, and western Junggar Mountains in Xinjiang. This species is also distributed in Central Asia, Southwest Asia, Western Siberia, and Central Europe. The three Gaoben-related medicinal materials are similar in morphology, resulting in the difficulty of distinguishing them from each other in appearance. DNA barcode ITS2, which was used to identify Gaoben medicinal materials and their adulterants, can distinguish adulterants from Gaoben, however, it was difficult to distinguish and identify its source plants *L. sinense* from *L. jeholense* (Gao et al., 2011).

Chloroplast (cp), an important organelle for photosynthesis in plants, is widely found in terrestrial plants, algae, and some protozoa. It is an important semiautonomous organelle in plant cells with its own genetic material and genetic system (Wolfe et al., 1987). In plants, the chloroplast genome has a conserved quadripartite structure comprising a large single-copy region (LSC) and a small single-copy (SSC) region, which are separated by a pair of inverted repeats (IRs). Most angiosperms have chloroplast genomes ranging in length from 120 to 160 kb (Chumley et al., 2006). Compared with mitochondrial and nuclear genes, chloroplast genomes are often used to explore the origin and evolution of plants, as well as plant genomics and bioinformatics due to their self-replication mechanism and relatively independent evolution (Daniell et al., 2016). The highly conserved structure with a moderate mutation rate of the chloroplast genome makes the comparative analysis suitable for phylogenetic studies at different taxonomic categories, including closely related species (Raubeson and Jansen, 2005; Yi and Choi, 2016).

Because of the different sources of Gaoben and Xinjiang Gaoben, the lack of systematic evaluation of alternative use brings great challenges to the safety and efficacy of clinical medication. In this study, we presented the complete chloroplast genomes of *L. sinense*, *L. jeholense*, and *C. vaginatum*. We aimed to (1) examine the variations including simple sequence repeats (SSRs) and other repeats among these three chloroplast genomes, and (2) discover highly divergent regions of the chloroplast genomes among the source plants of the medicinal materials Gaoben and Xinjiang Gaoben. This study provides beneficial information on the identification of Gaoben-related medicinal materials as well as further phylogenetic studies of Apiaceae.

## Materials and Methods

### Plant Material, DNA Extraction, and Sequencing

Fresh leaves of *L. sinense*, *L. jeholense*, and *C. vaginatum* were collected from Yuanbao village of Hubei, Qingyuan County of Liaoning, and Nilka County of Xijiang, respectively. All samples were identified by Professor Bengang Zhang, and Dr. Yaodong Qi at the Institute of Medicinal Plant Development (IMPLAD), the Chinese Academy of Medical Sciences (CAMS). The voucher specimens (GB2018052001, LGB2018052501, and XJGB2018080501) were deposited in the Research Center for Medicinal Plant Resources of IMPLAD. Total genomic DNA was extracted from the clean leaves frozen at −80°C using the Plant Genomic DNA Kit following the standard protocol (Tiangen Biotech Co., Ltd., Beijing). DNA quality was assessed based on spectrophotometry and electrophoresis in a 1% (w/v) agarose gel and a NanoDrop spectrophotometer 2000. High-quality DNA was used to generate shotgun libraries with an average insert size of 500 bp and sequenced using Illumina HiSeq X (v2, Illumina, San Diego CA, United States) in accordance with the standard protocol. Approximately, 10 GB of raw data was generated. After the fragments were filtered and trimmed by the fastp program, approximately 4 GB clean reads were obtained for each sample.

### Chloroplast Genome Assembly and Annotation

The high-quality paired-end reads were assembled by BLAST++, SSPACE_ Basic_ v2.0.pl. and SOAPdenovo-127mer (Wolfe et al., 1987; Camacho et al., 2009; Boetzer et al., 2011; Luo et al., 2012), and the cp genome of *Angelica gigas* (accession No.: KT963038) was chosen as the reference. Orientation and circularization of contigs using Geneious (Kearse et al., 2012). Finally, BWA was used to map clean reads to draft the chloroplast genome to ensure that each base was correct (Li and Durbin, 2009). Annotation was performed using the online tools CPGAVAS2 and GeSeq (Tillich et al., 2017; Shi et al., 2019). The genome maps were generated with the OGDRAW program (Lohse et al., 2013). The complete chloroplast genomes were deposited in GenBank with the accession numbers: *Ligusticum sinense* OM728579, *Ligusticum jeholense* OM728580, and *Conioselinum vaginatum* OM728581.

### Genome Structure Analysis and Genome Comparison

GC content was calculated using Geneious. The distribution of codon usage was investigated using CodonW with the RSCU ratio. SSRs were determined by using MISA. REPuter was used to identify and locate repeat sequences, including direct, reverse, palindromic, and complement repeats in the chloroplast genomes of three species. The minimal size was 30 bp and the two repeat copies had at least 90% similarity for all repeat types. MAFFT was used to compare the similarity of plastid genome sequences of the six species from **Apiaceae** (Katoh and Standley, 2013), and mVISTA was used to export visual results to evaluate similarity (Frazer et al., 2004). We also conducted a sliding window analysis to identify the nucleotide variability (Pi) of three Gaoben-related medicinal materials using DnaSP v5.10 (Librado and Rozas, 2009). The step size was set to 200 bp with an 800 bp window length (Zhou et al., 2018).

### Species Authentication Assessment

In order to evaluate the species authentication power of the two suggested highly variable regions according to sliding window analysis, we designed specific primers for each region. At the same time we also used two common barcode regions *psb*A-*trn*H and ITS2 for comparison (Supplementary Table S1). We collected five batches of medicinal slices from these three species, as well as one unknown species of *Ligusticum* from the medicine market respectively. We randomly selected three batches from three species and one batch of the unknown species for this study. Eight leaf samples from three species were collected from the field. All of the voucher medicinal materials and voucher specimens were deposited in the Research Center for Medicinal Plant Resources of IMPLAD (Supplementary Table S2). Total genomic DNA was extracted using the Plant Genomic DNA Kit following the standard protocol (Tiangen Biotech Co., Ltd., Beijing). PCR amplification of targeted DNA regions was performed using 12.5 μl 2 × EasyTaq PCR MasterMix (Biomed, Beijing, China), 1 μl each primer (5 μM), and 1–2 μl template DNA and finally replenished with distilled deionized water to the final volume of 25 μl. The purified PCR products were directly sequenced in both directions by Beijing Meijisinuo Biotec. Co., China. Sequences were assembled and aligned using Clustal X v.1.83 (Tompson et al., 1997) and BioEdit v.7.1.11 (Hall, 1999).

### Phylogenetic Analysis

In this study, *Panax notoginseng* was chosen as the outgroup. Chloroplast genomes of 31 species from Apiaceae were downloaded from GenBank (Supplementary Table S3). A total of 58 common protein-coding genes were extracted from 37 chloroplast genomes and the multiple sequences were then aligned using MAFFT v.7 (Katoh and Standley, 2013) and manually adjusted by MEGA 7.0 (Kumar et al., 2018). Phylogenetic analysis was conducted by maximum parsimony (MP), the maximum likelihood (ML), and the Bayesian inference (BI) respectively. MP analysis was carried out in PAUP * 4.0a with heuristic searches of 1,000 random stepwise sequence addition replicates and tree bisection-reconnection (TBR) branch swapping. Bootstrap values (MP-BS) were calculated with 1,000 pseudoreplicates of TBR branch swapping. Before ML and BI analysis, MrModeltest 2.4 was used to determine the best model. ML trees were reconstructed by performing a rapid bootstrap analysis on the RAxML web-server (Stamatakis, 2014), and the optimal nucleotide substitution model was GTR + I + G. BI analysis was carried out in the software package of MrBayes v3.2.1. Four Markov chains were run for 2,000,000 generations and were sampled every 1,000 generations with a random starting tree. After 25% ageing samples were discarded, a consistent tree was built according to the remaining samples, and a posteriori probability was calculated.

## Results and Discussion

### Features of the Chloroplast Genomes of Gaoben-Related Medicinal Materials

**Figure 2:**
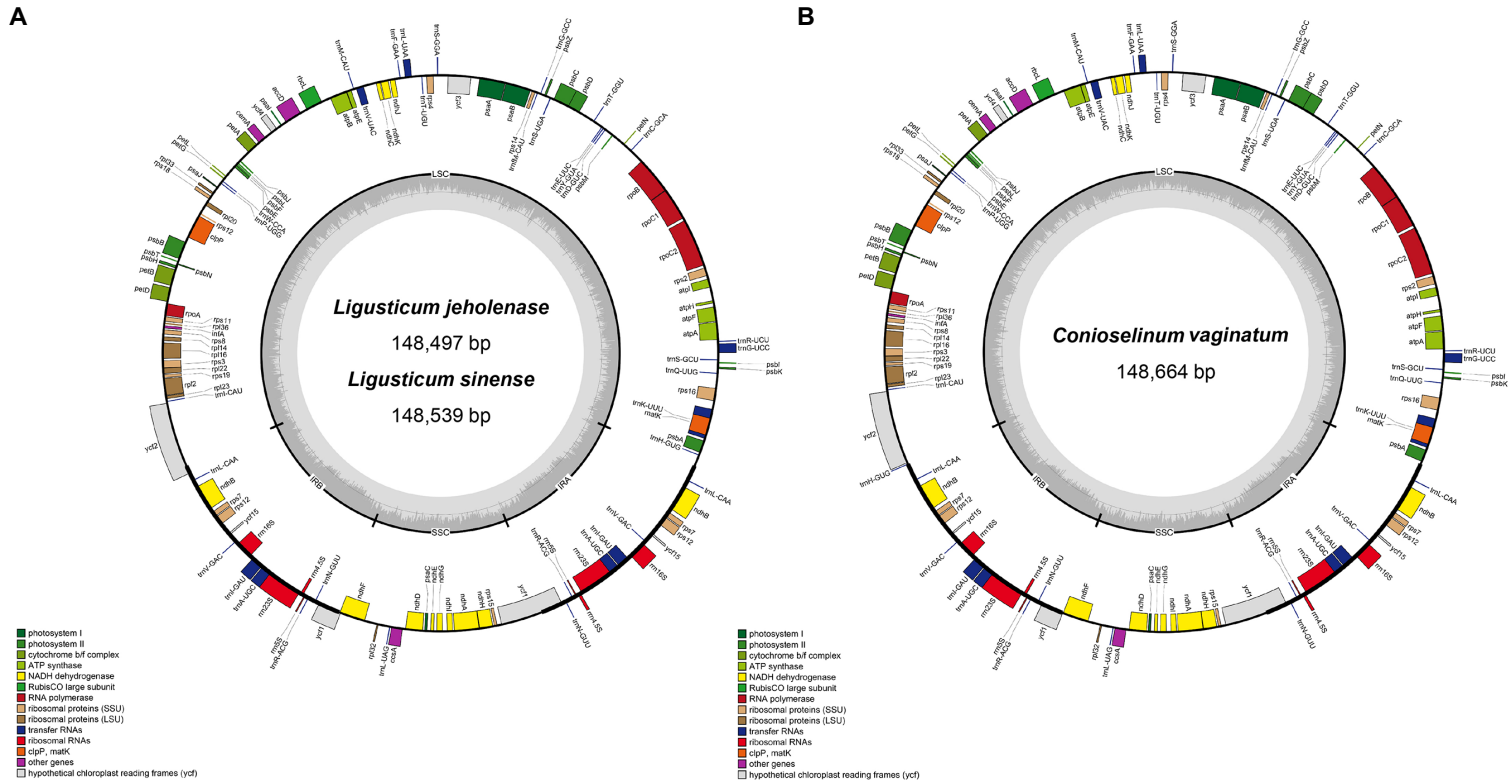
Chloroplast genome maps of **(A)** *Ligusticum sinense* and *Ligusticum jeholense*. **(B)** *Conioselinum vaginatum*. Genes drawn inside the circle are transcribed clockwise, and those outside the circle are transcribed counterclockwise. Dark grey shading in the inner circle represents the GC content.

The complete cp genomes of Gaoben-related medicinal materials obtained in this study exhibit a typical circular form with quadripartite structures (LSC, SSC, IRa, and IRb), whose total length was between 148.5–148.7 kb (Figure 2; Table 1). The longest one was *C. vaginatum*, 148,664 bp. The lengths of *L. sinense* and *L. jeholense* were 148,539 and 148,497 bp, respectively. The length of the IR region was between 18.5 and 18.6 kb. Among these species, *C. vaginatum*, with the longest cp genome, also had the longest IR region, and *L. jeholense* with the shortest cp genome, and had the shortest IR region. The length of the IR region was consistent with the total length of the chloroplast genome. The reverse repetition of IRa and IRb in the chloroplast genome makes the contraction and expansion of the IR region of most chloroplast genomes positively correlated with the total length.

**Table 1:** Base composition in the chloroplast genomes of *Conioselinum vaginatum*, *Ligusticum sinense*, and *Ligusticum jeholense*.

Species	Length/bp				GC content/%			
	Total	IR	LSC	SSC	Total	IR	LSC	SSC
*C. vaginatum*	148,664	18,613	93,777	17,661	37.61	44.93	35.96	30.99
*L. sinense*	148,539	18,477	93,978	17,607	37.61	44.82	35.99	31.12
*L. jeholense*	148,497	18,468	93,932	17,629	37.60	44.80	35.99	31.11

The GC content of both *C. vaginatum* and *L. sinense* was 37.61%, and that of *L. jeholense* was 37.6%. The GC content of the IR region of these three species was highest, with a range of 44.80 and 44.93%, and the SSC region was lowest, between with a range of 30.99 and 31.12% (Table 1). The high GC content in the IR detected here is similar to most previously reported cp genomes of the angiosperms (Asaf et al., 2020; Wei et al., 2020). The chloroplast genomes of these three species shared the same number and type of genes, and a total of 114 genes were identified, including 81 protein-coding genes (PCGs), 29 tRNA genes, and four rRNA genes. Of these, 15 genes were duplicated in the IR regions (Table 2). A total of 10 genes (*rps*12, rps16, *atp*F, *rpo*C1, *pet*B, *pet*D, *rpl*16, *rpl*2, *ndh*B, and *ndh*A) and six tRNA genes (*trn*K-UUU, *trn*G-UCC, *trn*L-UAA, *trn*V-UAC, *trn*I-GAU, and *trn*A-UGC) contained one intron, while two genes (*ycf*3, *clp*P) contained two introns.

**Table 2:** Gene contents in the chloroplast genomes of *C. vaginatum*, *L. sinense*, and *L. jeholense*.

Gene function	Classificaion of genes	Gene names
Genes for photosynthesis	Subunits of ATP synthase	*atp*A, *atp*B, *atp*E, *atp*F, *atp*H, *atp*I
	Subunits of NADH-dehydrogenase	*ndh*A, *ndh*B, *ndh*C, *ndh*D, *ndh*E, *ndh*F, *ndh*G, *ndh*H, *ndh*I, *ndh*J, *ndh*K
	Subunits of cytochrome b/f complex	*pet*A, *pet*B, *pet*D, *pet*G, *pet*L, *pet*N
	Subunits of photosystem I	*psa*A, *psa*B, *psa*C, *psa*I, *psa*J
	Subunits of photosystem II	*psb*A, *psb*B, *psb*C, *psb*D, *psb*E, *psb*F, *psb*H, *psb*I, *psb*J, *psb*K, *psb*L, *psb*M, *psb*N, *psb*T, *psb*Z
	Subunit of rubisco	*rbc*L
Other genes	Subunit of Acetyl-CoA-carboxylase	*acc*D
	c-type cytochrom synthesis gene	*ccs*A
	Envelop membrane protein	*cem*A
	Protease	*clp*P
	Translational initiation factor	*inf*A
	Maturase	*mat*K
Self-replication	Large subunit of ribosome	*rpl*16, *rpl*2, *rpl*14, *rpl*20, *rpl*22, *rpl*23, *rpl*32, *rpl*33, *rpl*36
	DNA dependent RNA polymerase	*rpo*A, *rpo*B, *rpo*C1, *rpo*C2
	Small subunit of ribosome	*rps*18, *rps*15, *rps*16, *rps*7, *rps*3, *rps*2, *rps*11, *rps*4, *rps*19, *rps*8, *rps*14, *rps*12, *rps*3
	rRNA Genes	*rrn*16S, *rrn*23S, *rrn*4.5S, *rrn*5S
	tRNA Genes	*trn*A-UGC, *trn*C-GCA, *trn*D-GUC, *trn*E-UUC, *trn*F-GAA, *trn*M-CAU, *trn*G-GCC, *trn*G-UCC, *trn*H-GUG, *trn*I-CAU, *trn*K-UUU, *trn*L-CAA, *trn*L-UAA, *trn*L-UAG, *trn*M-CAU, *trn*N-GUU, *trn*P-UGG, *trn*Q-UUG, *trn*R-ACG, *trn*R-UCU, *trn*S-GCU, *trn*S-GGA, *trn*S-UGA, *trn*T-GGU, *trn*T-UGU, *trn*V-GAC, *trn*V-UAC, *trn*W-CCA, *trn*Y-GUA
Genes of unkown function	Conserved open reading frames	*ycf*15, *ycf*1, *ycf*2, *ycf*3, *ycf*4

### Codon Usage Analysis

**Figure 3:**
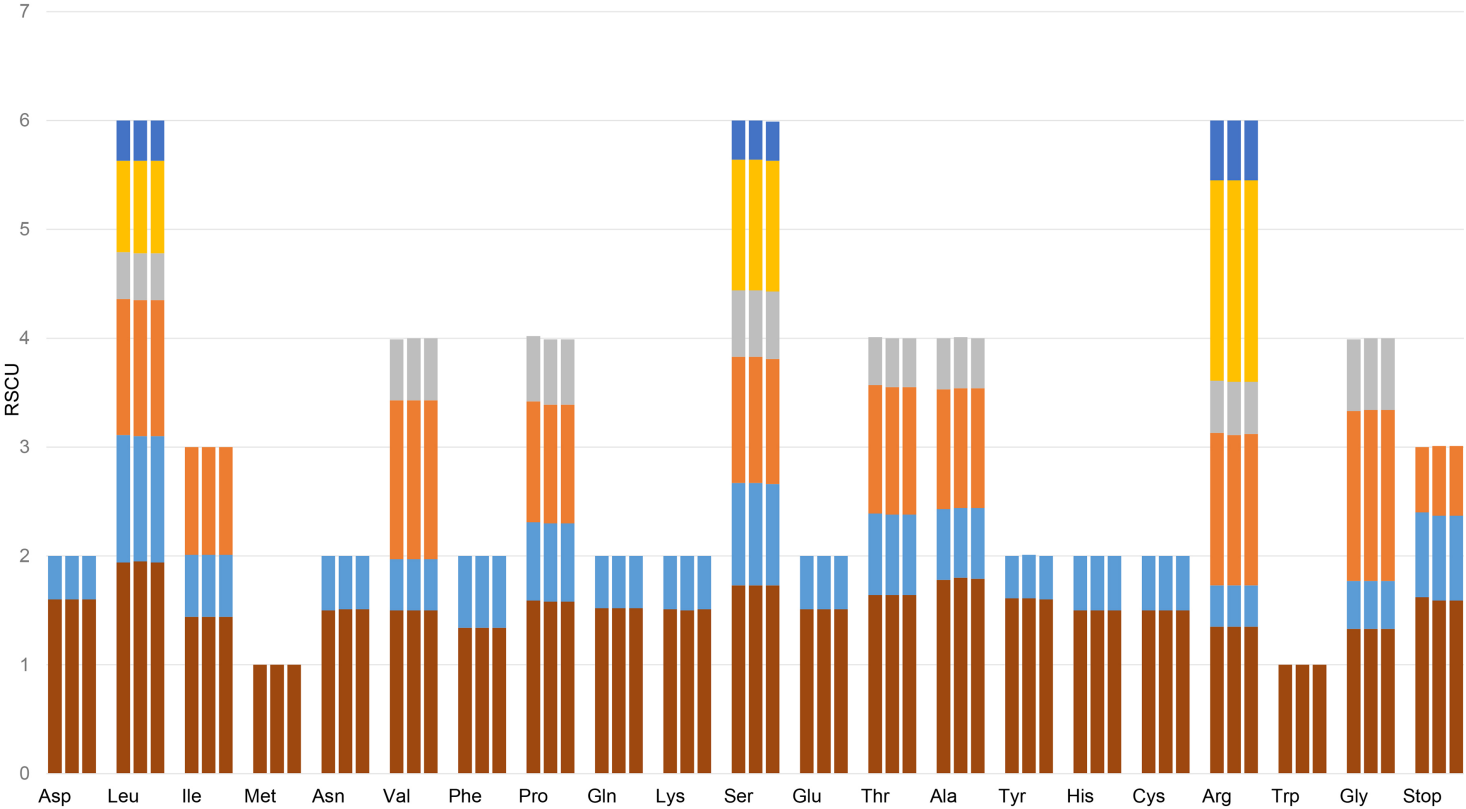
Codon content of 20 amino acid and stop codons in all protein-coding genes of the chloroplast genomes of three species. The histogram from left to right of each amino acid shows codon usage within the chloroplast genomes of *C. vaginatum*, *L. sinense*, and *L. jeholense*.

Codon usage in genomes tends to favor particular subsets of codons. Codon usage bias affects the translational speed and accuracy, and it is associated with the tRNA levels and the GC content in the genomes (Schmid and Flegel, 2011). Relative synonymous codon usage (RSCU) is a measure of nonuniform synonymous codon usage in coding sequences (Wang et al., 2018). Based on the sequences of protein-coding genes (CDSs), the codon usage frequency was estimated for the cp genomes of the three source plants of Gaoben-related medicinal material (Figure 3; Supplementary Table S4). The results revealed the presence of 64 codons, which encode 20 amino acids within the chloroplast protein-coding genes of these three species. All the protein-coding genes were composed of 24,320, 24,392, and 24,343 codons in the cp genomes of *C. vaginatum*, *L. sinense*, and *L. jeholense*, respectively. Leucine and cysteine were the most and least abundant universal amino acids in the cp genomes of the three species, respectively. Except methionine, amino acid codons in the chloroplast genomes of two species preferentially end with A or U (RSCU > 1). The third codon position was biased to end in A and U, accounting for 70.3% of all CDS codons, which is similar to most angiosperm cp genomes (Liu and Xue, 2005). RSCU value analysis showed that most of the amino acid codons have preferences, except methionine and tryptophan, whose codons exhibited no bias (RSCU = 1) in these three cp genomes. Seven codons were over-represented (RSCU > 1.6) in *C. vaginatum*, while five codons were over-represented in *L. sinense* and *L. jeholense*. Twenty codons were underrepresented (RSCU < 1.6) in all three species. High codon preference, especially a strong AT bias in codon usage, is much more common in other plant chloroplast genomes (Wu et al., 2010; Wang et al., 2016).

### Simple Sequence Repeats and Repeat Structure Analysis

Simple sequence repeats, also known as microsatellites, are abundantly distributed in the genome. SSRs comprise tandem repeated DNA sequences that consist of 1–6 repeat nucleotide units, and display a high level of polymorphism, placing them as the most preferred genetic markers for species identification, phylogenetic investigations, and other genetic studies (Powell et al., 1995; Yang et al., 2011; Jiao et al., 2012). A total of 84, 78, and 72 SSRs were detected in the cp genomes of *C. vaginatum*, *L. sinense*, and *L. jeholense*, respectively (Table 3), and the number of mononucleotides was between 40 (*L. jeholense*) and 47 (*C. vaginatum*). The chloroplast genome of Gaoben-related medicinal material contains 78 SSRs on average, including 44 mono-, 21 di-, 3 tri-, 7 tetra-, 2 penta-, and 1 hexa-nucleotide SSRs. The A/T mononucleotide repeats were the most common. TTA and ATTAG repeats in *C. vaginatum* were species-specific; AATA, AGATAT, and ATACTG existed in *L. sinense* and *L. jeholense*, while AATCA existed in *L. sinense* and *C. vaginatum*.

**Table 3:** Types and amounts of simple sequence repeats (SSRs) in the chloroplast genomes of *C. vaginatum*, *L. sinense*, and *L. jeholense*.

Species	SSR type						
	Mono	Di	Tri	Tetra	Penta	Hexa	Total
*C. vaginatum*	47	24	4	6	3	0	84
*L. sinense*	45	19	3	7	2	2	78
*L. jeholense*	40	19	3	7	1	2	72

Mono, mononucleotide; Di, dinucleotide; Tri, trinucleotide; Tetra, tetranucleotide; Penta, pentanucleotide; and Hexa, hexanucleotide.

**Figure 4:**
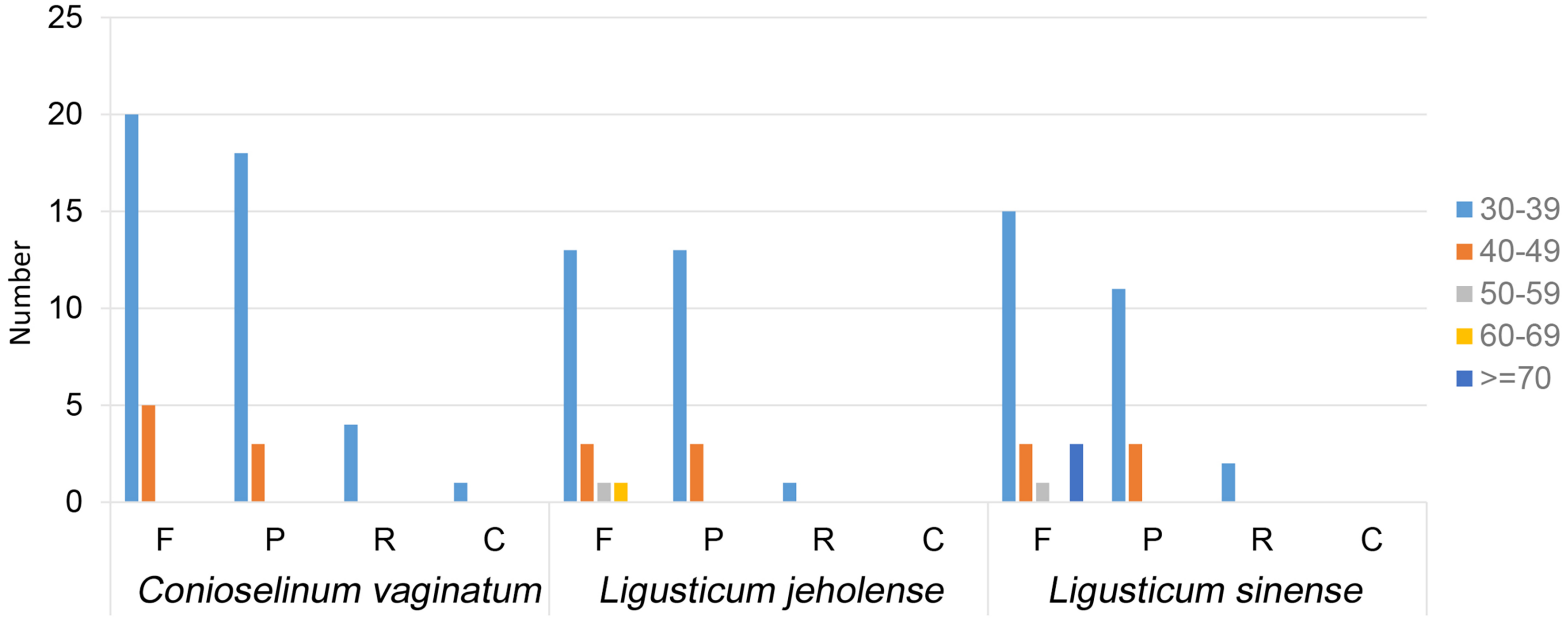
Repeat sequences in three chloroplast genomes. F, P, R, and C indicate the repeat types F (forward), P (palindrome), R (reverse), and C (complement), respectively. Repeats with different lengths are indicated in different colors.

Repeat structures are correlated with rearrangement and recombination of plastomes, which have been used in phylogenetic studies (Inkyu et al., 2017). Repeat sequences with a repeat unit longer than 30 bp were analyzed (Figure 4). The results revealed that the repeats of the chloroplast genome of *C. vaginatum* had the highest number of dispersed repeats, comprising 25 forwards, 21 palindromic, four reverse repeats, and one complement repeat. *L. jeholense* had the least dispersed repeats, which contained 18 forwards, 16 palindromic, and one reverse repeat. Most dispersed repeats were distributed in the intergenic regions. The majority of these repeats were mainly forwards and palindromic types with lengths mainly in the range of 30–50 bp. The repeat motif AATCT/AGATT in *C. vaginatum* was species-specific, ACTGAT/AGTATC and AGATAT/ATATCT existed in *L. sinense* and *L. jeholense*, while AAATC/ATTTG existed in *L. sinense* and *C. vaginatum*.

### Expansion and Contraction of the Border Regions

In the tetrad structure of the chloroplast genome, the IR region is more conserved than the SSC and LSC regions, but in the process of chloroplast evolution, the phenomenon of IR boundary contraction and expansion is widespread in plants (Zhou et al., 2018).

**Figure 5:**
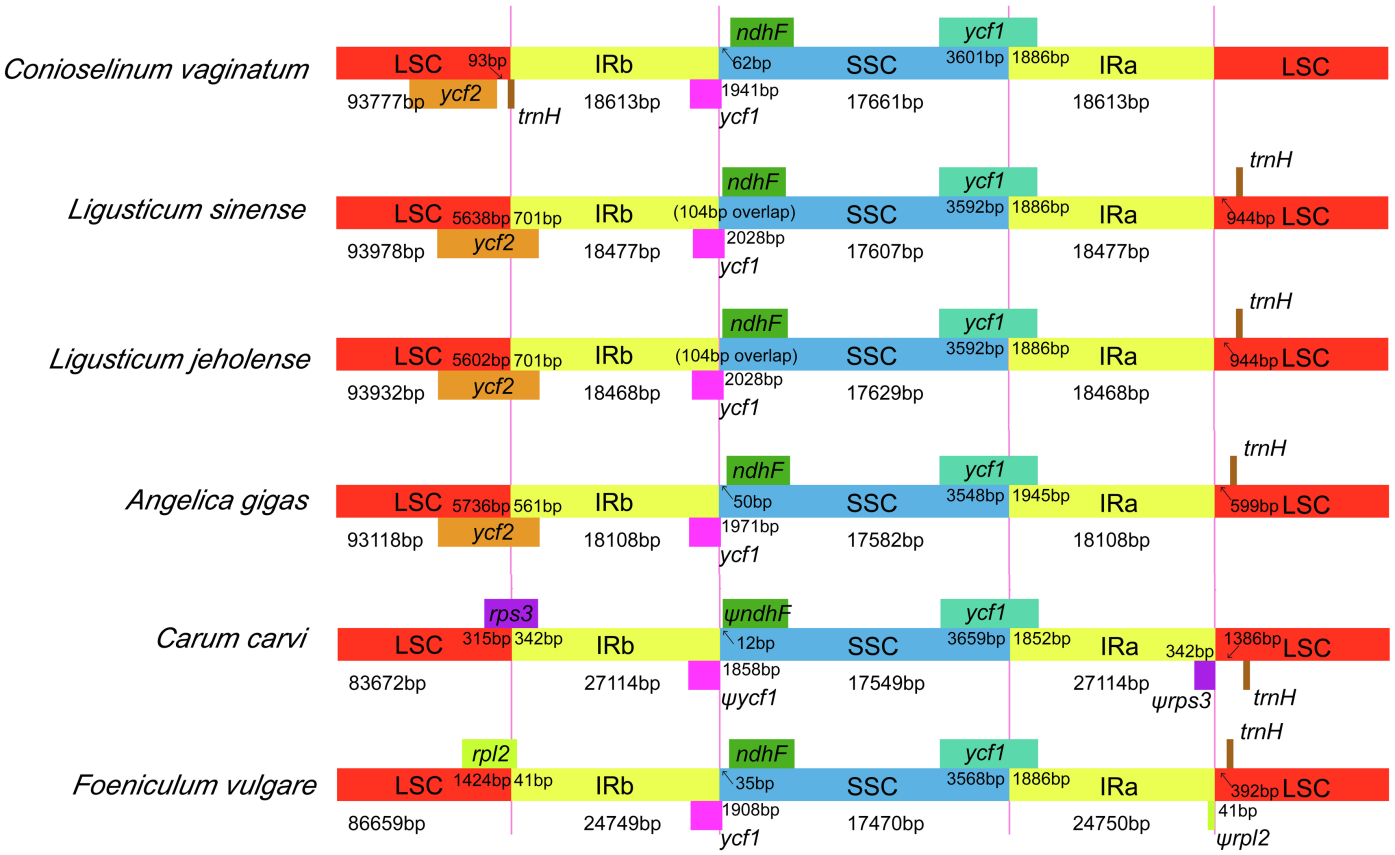
The borders of LSC, SSC, and IR regions among six chloroplast genomes. The number above the gene features means the distance between the ends of genes and the borders sites.

The border regions and adjacent genes of the cp genomes of the three Gaoben-related species were compared to analyze the expansion and contraction variation in junction regions. We selected three closely related species (*Carum carvi*, *Angelica gigas*, and *Foeniculum vulgare*) as references to compare the chloroplast genome structure (Figure 5). All of these species have the IRa/SSC boundary within the *ycf*1 gene and the IRb/SSC border between the *ycf*1 gene and ndhF or pseudogenes (ψ) *ycf*1 and (ψ) *ndh*F. IRb/LSC and IRa/LSC borders were obvious different in these species: the IRb/LSC boundary of *C. vaginatum* was in the *trn*H gene and there are 93 bp from *ycf*2 gene, while which of *L. sinense*, *L. jeholense*, and *Angelica gigas* were in *ycf*2 gene. The IRb/LSC boundary of *Carum carvi* and *Foeniculum vulgare* was in the *rps*3 and *rpl*2 genes, respectively.

### Comparative Genome Analysis

**Figure 6:**
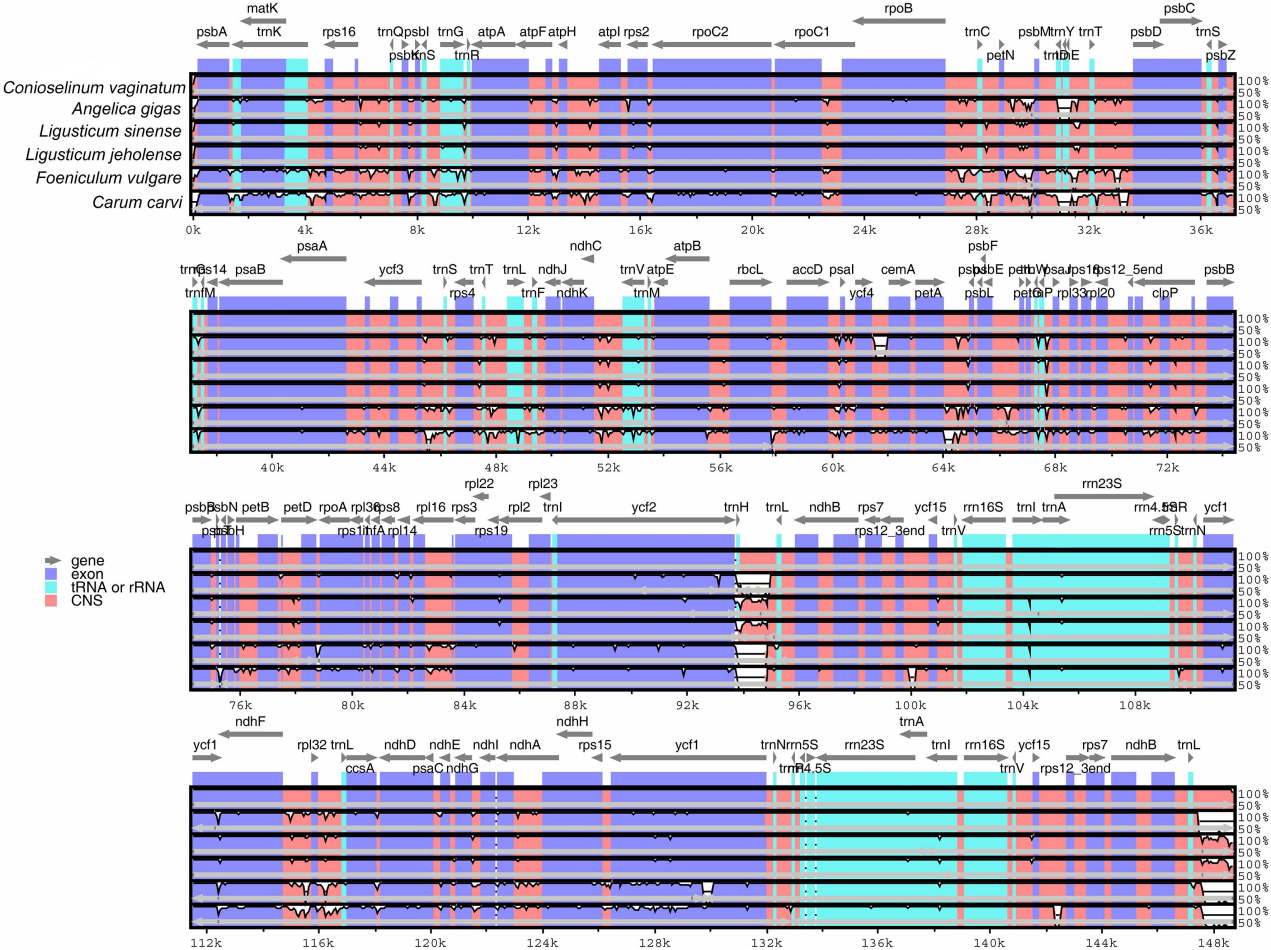
Sequence identity plot comparing the five chloroplast genomes with *Conioselinum vaginatum* as a reference by using mVISTA. Gray arrows and thick black lines above the alignment indicate genes with their orientation and the position of the inverted repeats (IRs), respectively. A cut-off of 70% identity was used for the plots, and the *Y*-scale represents the percent identity ranging from 50 to 100%.

The whole chloroplast genome sequences of *C. vaginatum*, *L. sinense*, and *L. jeholense* were compared with those of *C. carvi*, *A. gigas*, and *F. vulgare* using the mVISTA program (Figure 6). The chloroplast genomes of the above species are highly similar. The similarity of *L. sinense* and *L. jeholense* was higher, which showed obvious consistency in the positions of 6 k–10 kbp, 13 k–18 kbp, 28 k–34 kbp, 92 k–95 kbp, 115 k–117 kbp, and 147 k–149 bp. In general, the variation of the noncoding region was higher than the variation of the coding region and variation of the variation of the IR region was lower than the variation of the LSC and SSC regions.

**Figure 7:**
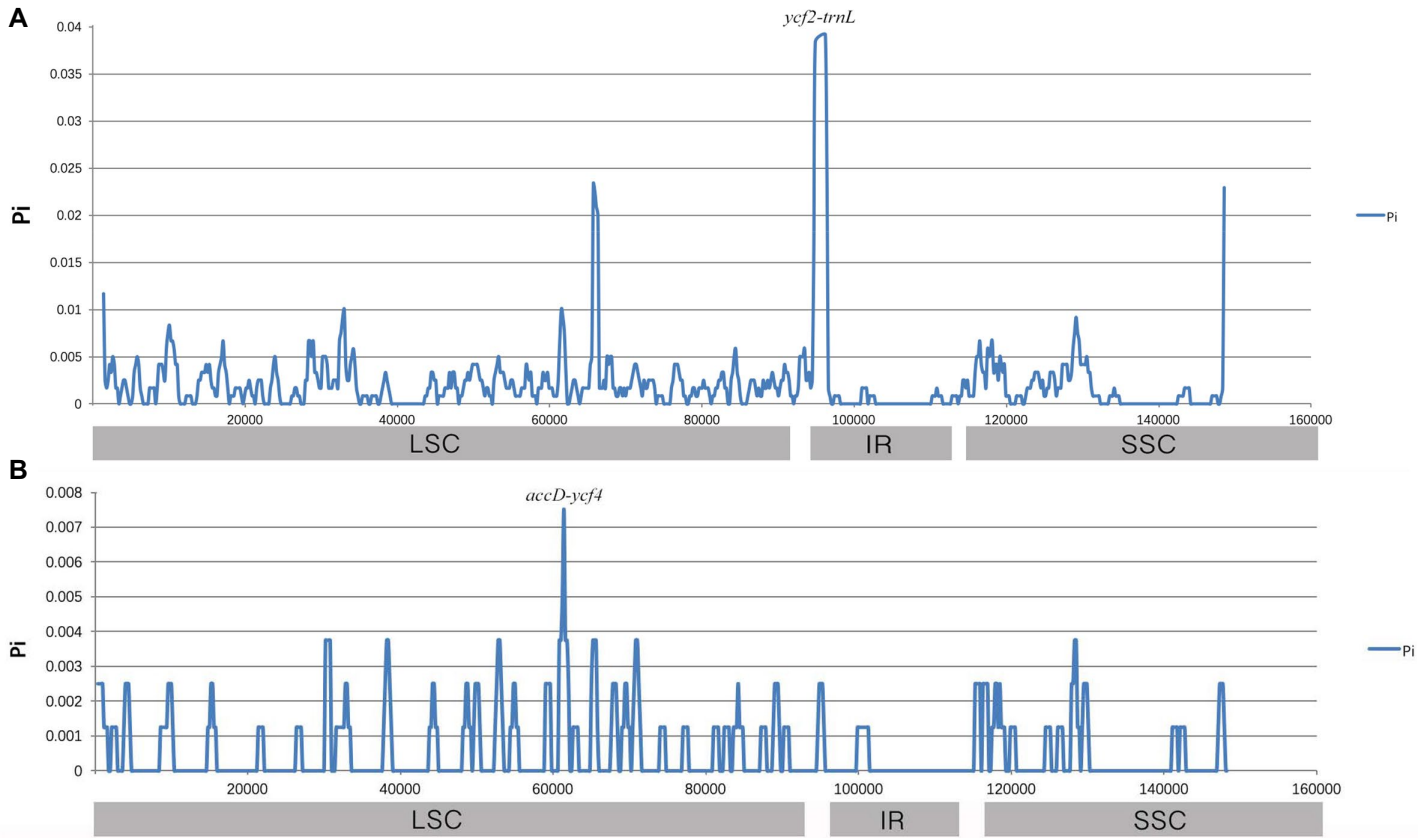
Sliding window analysis of the whole chloroplast genomes. Window length: 800 bp; step size: 200 bp. *X*-axis: position of the midpoint of a window. *Y*-axis: nucleotide diversity of each window. **(A)** Pi among *C. vaginatum*, *L. sinense*, and *L. jeholense*. **(B)** Pi between *L. sinense* and *L. jeholense*.

Furthermore, sliding window analysis using DnaSP detected highly variable regions in the chloroplast genomes of three Gaoben-related medicinal materials. Nucleotide variability (Pi) was calculated to show divergence at the sequence level (Figure 7). The average value of Pi was 0.002081 among the three Gaoben-related medicinal materials. The IR regions exhibited lower variability than the LSC and SSC regions. A mutational hotspot was observed, which showed remarkably higher P_i_ values (>0.035) and was located at the IR/LSC boundary. Comparing the nucleotide sequence of mutational hotspot, the variation hotspot was in *ycf*2-*trn*L due to *trn*H gene was inserted between *ycf*2 gene and *trn*L gene in *C. vaginatum*, while *trn*H gene in *L. sinense* and *L. jeholense* was not inserted in *ycf*2-*trn*L region, but located between *psb*A gene and *trn*L gene. These results suggested that this region might provide the scientific basis for distinguishing *C. vaginatum*, *L. sinense*, and *L. jeholense* (Figure 7A). The average value of P_i_ was 0.000573 between *L. sinense* and *L. jeholense*. The variation hotspot which showed remarkably higher P_i_ values (>0.007) was located at in LSC regions (Figure 7B). Comparing the nucleotide sequence, the variation hotspot was in the *acc*D-*ycf*4 region, in which region, there were three SNPs between *L. sinense* and *L. jeholense*, and one 7 bp indel (ATAATAA) between *C. vaginatum* and *L. sinense*, and *L. jeholense* (Table 4).

**Table 4:** Variation of *acc*D*-ycf*4 in *L. sinense*, *L. jeholense*, and *C. vaginatum*.

Species	*acc*D*-ycf*4			
	247	278	318	397–403
*L. sinense*	A	C	C	-
*L. jeholense*	G	A	T	-
*C. vaginatum*	A	C	C	ATAATAA

### Species Authentication

Two highly variable regions *ycf*2-*trn*L and *acc*D-*ycf*4 as well as two common barcode regions *psb*A-*trn*H and ITS2 were amplified and sequenced successfully in both medicinal slices and leaf samples (Supplementary Figure S1). The sequence length of *C. vaginatum* was significantly longer than that of *L. sinense* and *L. jeholense* because of the insertion of *trn*H gene into *ycf*2-*trn*L in *C. vaginatum*. Sequences of *C. vaginatum* were 925 bp longer than of *L. sinense* and *L. jeholense*. One stability SNP at loci 152 (A/G) of *ycf*2-*trn*L can distinguish *L. sinense* and *L. jeholense* (Supplementary Data). The 7 bp indel (ATAATAA) at loci 397–403 of *acc*D*-ycf*4 was stable between *C. vaginatum* and *L. sinense*, *L. jeholense* and can be used to distinguish *C. vaginatum*. Only one SNP at loci 274 were stable exist between *L. sinense* and *L. jeholense*, intraspecific variation was found in the other two SNPs recognized by sliding window analysis (Supplementary Data). The sequences of unknown species from the medicine market were identified as *Ligusticum* but the species was still uncertain. Previous studies pointed out that the commonly used DNA barcodes ITS2, *psb*A-*trn*H and *acc*D were useful in distinguishing *L. jeholense* from adulterants from other genera but not from the same genus (Gao et al., 2011; Li et al., 2015). In our study, the results showed the similar results that ITS2 and *psb*A-*trn*H could separate *C. vaginatum* from *L. sinense* and *L. jeholense*. *Ligusticum sinense* and *L. jeholense* had two SNPs in ITS2, but no difference in *psb*A-*trn*H (Supplementary Data). Our study suggests that *ycf*2-*trn*L and *acc*D-*ycf*4 could be effective barcodes for distinguishing the three Gaoben-related medicinal materials *C. vaginatum*, *L. sinense*, and *L. jeholense*. Especially, *ycf*2-*trn*L can distinguish *C. vaginatum* from the others based on the results of gel electrophoresis without sequencing. In addition, this study further revealed that the high-variable regions suggested by comparative cp genome analysis can be used as potential molecular markers for species authentication only after verification.

### Phylogenetic Analysis

**Figure 8:**
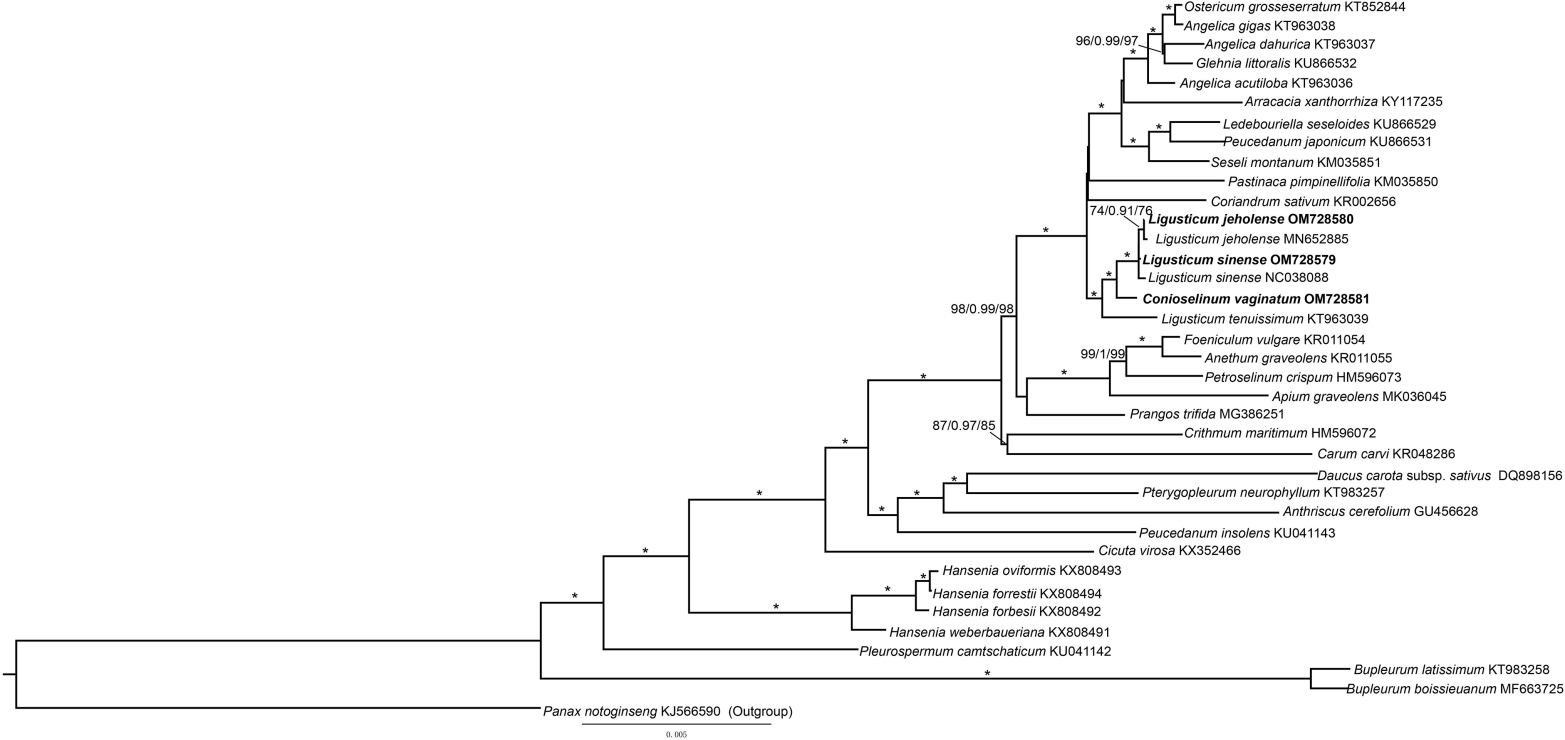
Phylogenetic tree of Apiaceae obtained from the maximum likelihood (ML) analysis of chloroplast genomes. Numbers on branches are support values (BS_ML_/PP_BI_/BS_MP_), Stars indicate BS_MP_, BS_ML_ = 100%, and PP_BI_ = 1.0.

To study the phylogeny of three species of Gaoben-related medicinal material, the protein coding region sequence of the chloroplast genome of 31 species of Apiaceae was analyzed, and *Panax notoginseng* was selected as the outgroup. The three phylogenetic analyses (MP, ML, and BI) revealed congruent topologies. Three species of *Ligusticum* and *C. vaginatum* were recognized as well-supported monophyly (Figure 8, BB_MP_ = 100, BB_ML_ = 100, and PP_BI_ = 1). *Ligusticum sinense* and *L. jeholense* gathered into a sister group with *C. vaginatum*, and then with *L. tenuissimum*, they formed a sister group. The pervious phylogenetic study of *Ligusticum* inferred from cp genomes and ITS showed that *Ligusticum* was not monophyly but mixed with others species from *Acronema*, *Sinodielsia*, and *Selineae* (Ren et al., 2020). Our study revealed that *Conioselinum* embedded *Ligusticum* which is consistent with the previous phylogenetic studies of the subfamily Apioideae that *Ligusticum* and *Conioselinum* were clearly not monophyletic. These two genera are both in need of revision (Downie et al., 2000). Extensive sampling and more robust molecular data are necessary to understand the phylogenetic relationships within Apiaceae (Wen et al., 2020).

## Conclusion

The complete chloroplast genomes of three Gaoben-related medicinal materials were determined in this study. The results revealed that genome size, structure, gene content, as well as compositional organization were highly conserved among the three species. Species of *Ligusticum* and *C. vaginatum* were recognized as well-supported monophyly, which revealed the closely related relationship between *Ligusticum* and *Conioselinum*. Our study suggested that *ycf*2-*trn*L and *acc*D-*ycf*4 could be effective barcodes for distinguishing the three Gaoben-related medicinal materials *C. vaginatum*, *L. sinense*, and *L. jeholense*. This study provided a new method for the accurate identification of three Gaoben-related medicinal materials and provided a scientific basis for differential evaluation of Gaoben-related medicinal materials.

## Data Availability Statement

The complete cp genome sequences of *Ligusticum sinense*, *Ligusticum jeholense*, and *Conioselinum vaginatum* are deposited in GenBank, accession number: OM728579, OM728580, and OM728581.

## Author Contributions

X-PW was responsible for data analysis and writing of the manuscript. X-YZ and Y-QD performed the bioinformatics work. X-PW, Y-JB, and J-LC did experimental work and data analyses. J-SL participated in the production of figures. Y-DQ and B-GZ collected and identified samples. H-TL conceived the design of the study. All authors contributed to the article and approved the submitted version.

## Funding

This research was funded by National Natural Science Foundation of China (Grant No. 81673543) and the State Key R&D Program on “Modernization of Traditional Chinese Medicine” (2018YFC1707904).

## Conflict of Interest

The authors declare that the research was conducted in the absence of any commercial or financial relationships that could be construed as a potential conflict of interest.

## Publisher’s Note

All claims expressed in this article are solely those of the authors and do not necessarily represent those of their affiliated organizations, or those of the publisher, the editors and the reviewers. Any product that may be evaluated in this article, or claim that may be made by its manufacturer, is not guaranteed or endorsed by the publisher.
