# Supplementary material for: Molecular Structure and Phylogenetic Analyses of the Complete Chloroplast Genomes of Three Medicinal Plants Conioselinum vaginatum, Ligusticum sinense, and Ligusticum jeholense
Source: Front Plant Sci. 2022 Jun 6;13:878263. doi: 10.3389/fpls.2022.878263 (PMC9207526; doi:10.3389/fpls.2022.878263)
Supplement: Supplementary file 1 [file Data_Sheet_1.PDF]

>1Ligusticum_sinense_accD_ycf4
AGAGGCCTCTATCAACAAGAAAAATAGTGAATTCTTATTTTCGTTAAATTCTAGGAAAAT
AAAAAATTTTTGTTCTACGTCTTACGTATGTATATATAATCCAAATATAGAATTCTAGAA
TATAGAAACTATAGATATGATATATGCTTTTGTACCTTCTATACTCACTTAGATATATAC
TTAGATTTATACTAATCTTTATCTTTATAATATTAATATATATATATAAATAATAA------
-TAACAGGTACAAATAGTAAATTGAGGTATCCTTTATATGACA
>2Ligusticum_sinense_accD_ycf4
AGAGGCCTCTATCAACAAGAAAAATAGTGAATTCTTATTTTCGTTAAATTCTAGGAAAAT
AAAAAATTTTTGTTCTACGTCTTACGTATGTATATATAATCCAAATATAGAATTCTAGAA
TATAGAAACTATAGATATGATATATGCTTTTGTACCTTCTATACTCACTTAGATATATAC
TTAGATTTATACTAATCTTTATCTTTATAATATTAATATATATATATAAATAATAA------
-TAACAGGTACAAATAGTAAATTGAGGTATCCTTTATATGACA
>3Ligusticum_sinense_accD_ycf4
AGAGGCCTCTATCAACAAGAAAAATAGTGAATTCTTATTTTCGTTAAATTCTAGGAAAAT
AAAAAATTTTTGTTCTACGTCTTACGTATGTATATATAATCCAAATATAGAATTCTATAA
TATAGAAACTATAGATATGATATATGCTTTTGTACCTTCTATACTCACTTAGATATATAC
TTAGATTTATACTAATCTTTATCTTTATAATATTAATATATATATATAAATAATAA------
-TAACAGGTACAAATAGTAAATTGAGGTATCCTTTATATGACA
>4Ligusticum_jeholense_accD_ycf4
AGAGGCCTCTATCAACAAGAAAAATAGTGAATTCTTATTTTCGTTAAATTCTAGGAAAAT
AAAAAATTTTTGTTCTACGTCTTGCGTATGTATATATAATCCAAATATAGAATTCTAGAA
TATAGAAACTATAGATATGATATATGCTTTTGTACCTTCTATACTCACTTAGATATATAC
TTAGATTTATACTAATCTTTATCTTTATAATATTAATATATATATATAAATAATAA------
-TAACAGGTACAAATAGTAAATTGAGGTATCCTTTATATGACA
>5Ligusticum_jeholense_accD_ycf4
AGAGGCCTCTATCAACAAGAAAAATAGTGAATTCTTATTTTCGTTAAATTCTAGGAAAAT
AAAAAATTTTTGTTCTACGTCTTGCGTATGTATATATAATCCAAATATAGAATTCTAGAA
TATAGAAACTATAGATATGATATATGCTTTTGTACCTTCTATACTCACTTAGATATATAC
TTAGATTTATACTAATCTTTATCTTTATAATATTAATATATATATATAAATAATAA------
-TAACAGGTACAAATAGTAAATTGAGGTATCCTTTATATGACA
>6Ligusticum_jeholense_accD_ycf4
AGAGGCCTCTATCAACAAGAAAAATAGTGAATTCTTATTTTCGTTAAATTCTAGGAAAAT
AAAAAATTTTTGTTCTACGTCTTGCGTATGTATATATAATCCAAATATAGAATTCTAGAA
TAAAGAAACTATAGATATGATATATGCTTTTGTACCTTCTATACTCACTTAGATATATAC
TTAGATTTATACTAATCTTTATCTTTATAATATTAATATATATATATAAATAATAA------
-TAACAGGTACAAATAGTAAATTGAGGTATCCTTTATATGACA
>12Ligusticum_jeholense_accD_ycf4
AGAGGCCTCTATCAACAAGAAAAATAGTGAATTCTTATTTTCGTTAAATTCTAGGAAAAT
AAAAAATTTTTGTTCTACGTCTTGCGTATGTATATATAATCCAAATATAGAATTATAGAA
TATAGAAACTATAGATATGATATATGCTTTTGTACCTTCTATACTCACTTAGATATATAC
TTAGATTTATACTAATCTTTATCTTTATAATATTAATATATATATATAAATAATAA------
-TAACAGGTACAAATAGTAAATTGAGGTATCCTTTATATGACA
>13Ligusticum_jeholense_accD_ycf4
AGAGGCCTCTATCAACAAGAAAAATAGTGAATTCTTATTTTCGTTAAATTCTAGGAAAAT
AAAAAATTTTTGTTCTACGTCTTGCGTATGTATATATAATCCAAATATAGAATTCTAGAA
TATAGAAACTATAGATATGATATATGCTTTTGTACCTTCTATACTCACTTAGATATATAC
TTAGATTTATACTAATCTTTATCTTTATAATATTAATATATATATATAAATAATAA------
-TAACAGGTACAAATAGTAAATTGAGGTATCCTTTATATGACA
>14Ligusticum_jeholense_accD_ycf4
AGAGGCCTCTATCAACAAGAAAAATAGTGAATTCTTATTTTCGTTAAATTCTAGGAAAAT
AAAAAATTTTTGTTCTACGTCTTGCGTATGTATATATAATCCAAATATAGAATTCTAGAA
TATAGAAACTATAGATATGATATATGCTTTTGTACCTTCTATACTCACTTAGATATATAC
TTAGATTTATACTAATCTTTATCTTTATAATATTAATATATATATATAAATAATAA------

-TAACAGGTACAAATAGTAAATTGAGGTATCCTTTATATGACA
>18Ligusticum_jeholense_accD_ycf4
AGAGGCCTCTATCAACAAGAAAAATAGTGAATTCTTATTTTCGTTAAATTCTAGGAAAAT
AAAAAATTTTTGTTCTACGTCTTGCGTATGTATATATAATCCAAATATAGAATTCTAGAA
TATAGAAACTATAGATATGATATATGCTTTTGTACCTTCTATACTCACTTAGATATATAC
TTAGATTTATACTAATCTTTATCTTTATAATATTAATATATATATAAATAATAA------
-TAACAGGTACAAATAGTAAATTGAGGTATCCTTTATATGACA
>7Conioselinum_vaginatum_accD_ycf4
AGAGGCCTCTATCAACAAGAAAAATAGTGAATTCTTATTTTCGTTAAATTCTAGGAAAAT
AAAAAATTTTTGTTCTACGTCTTACGTATGTATATATAATCCAAATATAGAATTCTAGAA
TATAGAAACTATAGATATGATATATGCTTTTGTACCTTCTATACTCACTTAGATATATAC
TTAGATTTATACTAATCTTTATCTTTATAATATTAATATATATATAAATAATAAATAATA
ATAACAGGTACAAATAGTAAATTGAGGTATCCTTTATATGACA
>8Conioselinum_vaginatum_accD_ycf4
AGAGGCCTCTATCAACAAGAAAAATAGTGAATTCTTATTTTCGTTAAATTCTAGGAAAAT
AAAAAATTTTTGTTCTACGTCTTACGTATGTATATATAATCCAAATATAGAATTCTAGAA
TATAGAAACTATAGATATGATATATGCTTTTGTACCTTCTATACTCACTTAGATATATAC
TTAGATTTATACTAATCTTTATCTTTATAATATTAATATATATATAAATAATAAATAATA
ATAACAGGTACAAATAGTAAATTGAGGTATCCTTTATATGACA
>9Conioselinum_vaginatum_accD_ycf4
AGAGGCCTCTATCAACAAGAAAAATAGTGAATTCTTATTTTCGTTAAATTCTAGGAAAAT
AAAAAATTTTTGTTCTACGTCTTACGTATGTATATATAATCCAAATATAGAATTCTAGAA
TATAGAAACTATAGATATGATATATGCTTTTGTACCTTCTATACTCACTTAGATATATAC
TTAGATTTATACTAATCTTTATCTTTATAATATTAATATATATATAAATAATAAATAATA
ATAACAGGTACAAATAGTAAATTGAGGTATCCTTTATATGACA
>15Conioselinum_vaginatum_accD_ycf4
AGAGGCCTCTATCAACAAGAAAAATAGTGAATTCTTATTTTCGTTAAATTCTAGGAAAAT
AAAAAATTTTTGTTCTACGTCTTACGTATGTATATATAATCCAAATATAGAATTCTAGAA
TATAGAAACTATAGATATGATATATGCTTTTGTACCTTCTATACTCACTTAGATATATAC
TTAGATTTATACTAATCTTTATCTTTATAATATTAATATATATATAAATAATAAATAATA
ATAACAGGTACAAATAGTAAATTGAGGTATCCTTTATATGACA
>16Conioselinum_vaginatum_accD_ycf4
AGAGGCCTCTATCAACAAGAAAAATAGTGAATTCTTATTTTCGTTAAATTCTAGGAAAAT
AAAAAATTTTTGTTCTACGTCTTACGTATGTATATATAATCCAAATATAGAATTCTAGAA
TATAGAAACTATAGATATGATATATGCTTTTGTACCTTCTATACTCACTTAGATATATAC
TTAGATTTATACTAATCTTTATCTTTATAATATTAATATATATATAAATAATAAATAATA
ATAACAGGTACAAATAGTAAATTGAGGTATCCTTTATATGACA
>17Conioselinum_vaginatum_accD_ycf4
AGAGGCCTCTATCAACAAGAAAAATAGTGAATTCTTATTTTCGTTAAATTCTAGGAAAAT
AAAAAATTTTTGTTCTACGTCTTACGTATGTATATATAATCCAAATATAGAATTCTAGAA
TATAGAAACTATAGATATGATATATGCTTTTGTACCTTCTATACTCACTTAGATATATAC
TTAGATTTATACTAATCTTTATCTTTATAATATTAATATATATATAAATAATAAATAATA
ATAACAGGTACAAATAGTAAATTGAGGTATCCTTTATATGACA
