# Supplementary material for: Molecular Structure and Phylogenetic Analyses of the Complete Chloroplast Genomes of Three Medicinal Plants Conioselinum vaginatum, Ligusticum sinense, and Ligusticum jeholense
Source: Front Plant Sci. 2022 Jun 6;13:878263. doi: 10.3389/fpls.2022.878263 (PMC9207526; doi:10.3389/fpls.2022.878263)
Supplement: Supplementary file 2 [file Data_Sheet_2.PDF]

>1Ligusticum_sinense_ITS2
TTATGCGATTA-CTGGTGTGAATTGCAGAATCCCGTGAACCATCGAGTCTTTGAACGCAA
GTTGCGCCCGAAGCCATTAGGCTGAGGGCACGTCTGCCTGGGTGTCACGCATCATCTTTG
CCAACAACCACACACTCCTTCAAGAGCTGTGCCGGTTTGGGGCGGAAATTGGCCTCCCGT
GCCTTGTTGTGCGGTTGGTGCAAAAGTGAGTCTCCGGCGACGGACGTCGTGACATCGGTG
GTTGTAAAAGACCCTCATGTCTTGTCACGCGAATCTGCGTCATCTTAGTGAGCTCTAGGA
CCCCTAGGCAGCACACACTCTGTGCGCTTCGATTGTGACCCCAGGTCAGGCGGGACTACC
CGCTGAGTTTAAGCATATCAATAAGCGGAGGAAAAGAAACTTACAAGGATTCCCCTAGTA
ACGGCGAGCGAACCGGGAACAGCCCAGCTTGAAAATTGGGCGGCTTTGCCGTCTGAATTG
TAGTCTGAGGAAAAGCGTCA
>2Ligusticum_sinense_ITS2
TTATGCGATTA-CTGGTGTGAATTGCAGAATCCCGTGAACCATCGAGTCTTTGAACGCAA
GTTGCGCCCGAAGCCATTAGGCTGAGGGCACGTCTGCCTGGGTGTCACGCATCATCTTTG
CCAACAACCACACACTCCTTCAAGAGCTGTGCCGGTTTGGGGCGGAAATTGGCCTCCCGT
GCCTTGTTGTGCGGTTGGTGCAAAAGTGAGTCTCCGGCGACGGACGTCGTGACATCGGTG
GTTGTAAAAGACCCTCATGTCTTGTCACGCGAATCTGCGTCATCTTAGTGAGCTCTAGGA
CCCCTAGGCAGCACACACTCTGTGCGCTTCGATTGTGACCCCAGGTCAGGCGGGACTACC
CGCTGAGTTTAAGCATATCAATAAGCGGAGGAAAAGAAACTTACAAGGATTCCCCTAGTA
ACGGCGAGCGAACCGGGAACAGCCCAGCTTGAAAATTGGGCGGCTTTGCCGTCTGAATTG
TAGTCTGAGGAAA-GCGTCA
>10Ligusticum_sp_ITS2
TTATGCGATTA-CTGGTGTGAATTGCAGAATCCCGTGAACCATCGAGTCTTTGAACGCAA
GTTGCGCCCGAAGCCATTAGGCTGAGGGCACGTCTGCCTGGGTGTCACGCATCATCTTTG
CCAACAACCACACACTCCTTCAAGAGCTGTGCCGGTTTGGGGCGGAAATTGGCCTCCCGT
GCCTTGTTGTGCGGTTGGTACAAAAGTGAGTCTCCGGCGACGGACGTCGTGACATCGGTG
GTTGTAAAAGACCCTCATGTCTTGTCACGCGAATCTGCGTCATCTTAGTGAGCTCTAGGA
CCCCTAGGCAGCACACACTCTGTGCGCTTCGATTGTGACCCCAGGTCAGGCGGGACTACC
CGCTGAGTTTAAGCATATCAATAAGCGGAGGAAAAGAAACTTACAAGGATTCCCCTAGTA
ACGGCGAGCGAACCGGGAACAGCCCAGCTTGAAAATTGGGCGGCTTTGCCGTCTGAATTG
TAGTCTGAGGAAAGCGTCA
>11Ligusticum_sinense_ITS2
TTATGCGATTA-CTGGTGTGAATTGCAGAATCCCGTGAACCATCGAGTCTTTGAACGCAA
GTTGCGCCCGAAGCCATTAGGCTGAGGGCACGTCTGCCTGGGTGTCACGCATCATCTTTG
CCAACAACCACACACTCCTTCAAGAGCTGTGCCGGTTTGGGGCGGAAATTGGCCTCCCGT
GCCTTGTTGTGCGGTTGGTGCAAAAGTGAGTCTCCGGCGACGGACGTCGTGACATCGGTG
GTTGTAAAAGACCCTCATGTCTTGTCACGCGAATCTGCGTCATCTTAGTGAGCTCTAGGA
CCCCTAGGCAGCACACACTCTGTGCGCTTCGATTGTGACCCCAGGTCAGGCGGGACTACC
CGCTGAGTTTAAGCATATCAATAAGCGGAGGAAAAGAAACTTACAAGGATTCCCCTAGTA
ACGGCGAGCGAACCGGGAACAGCCCAGCTTGAAAATTGGGCGGCTTTGCCGTCTGAATTG
TAGTCTGGAGAAAAGCGTCA
>12Ligusticum_jeholense_ITS2
TTATGCGATTA-CTGGTGTGAATTGCAGAATCCCGTGAACCATCGAGTCTTTGAACGCAA
GTTGCGCCCGAAGCCATTAGGCTGAGGGCACGTCTGCCTGGGTGTCACGCATCATCTTTG
CCAACAACCACACACTCCTTCAAGAGCTGTGCCGGTTTGGGGCGGAAATTGGCCTCCCGT
GCCTTGTTGTGCGGTTGGTGCAAAAGTGAGTCTCCGGCGACGGACGTCGTGACATCGGTG
GTTGTAAAAGACCCTCATGTCTTGTCACGCGAATCTGCGTCATCTTAGTGAGCTCTAGGA
CCCCTAGGCGGCACACACTCTGTGCGCTTCGATTGTGACCCCAGGTCAGGCGGGACTACC
CGCTGAGTTTAAGCATATCAATAAGCGGAGGAAAAGAAACTTACAAGGATTCCCCTAGTA
ACGGCGAGCGAACCGGGAACAGCCCAGCTTGAAAATTGGGCGGCTTTGCCGTCTGAATTG
TAGTCTGGGAAAGGCGTCA
>4Ligusticum_jeholense_ITS2
-TATGCGATTTCCTGGTGTGAATTGCAGAATCCCGTGAACCATCGAGTCTTTGAACGCAA
GTTGCGCCCGAAGCCATTAGGCTGAGGGCACGTCTGCCTGGGTGTCACGCATCATCTTTG

CCAACAACCACACACTCCTTCAAGAGCTGTGCCGGTTTGGGGCGGAAATTGGCCTCCCGT
GCCTTGTTGTGCGGTTGGCGCAAAAGCGAGTCTCCGGCGACGGACGTCGTGACATCGGTG
GTTGTAAAAGACCCTCATGTCTTGTCACGCGAATCTGCGTCATCTTAGTGAGCTCTAGGA
CCCCTAGGCAGCACACACTCTGTGCGCTTCGATTGTGACCCCAGGTCAGGCGGGACTACC
CGCTGAGTTTAAGCATATCAATAAGCGGAGGAAAAGAAACTTACAAGGATTCCCCTAGTA
ACGGCGAGCGAACCGGGAACAGCCCAGCTTGAAAATTGGGCGGCTTTGCCGTCTGAATTG
TAGTCTGAGGAAA-GCGTCA
>5Ligusticum_jeholense_ITS2
TTATGCGATTA-CTGGTGTGAATTGCAGAATCCCGTGAACCATCGAGTCTTTGAACGCAA
GTTGCGCCCGAAGCCATTAGGCTGAGGGCACGTCTGCCTGGGTGTCACGCATCATCTTTG
CCAACAACCACACACTCCTTCAAGAGCTGTGCCGGTTTGGGGCGGAAATTGGCCTCCCGT
GCCTTGTTGTGCGGTTGGCGCAAAAGCGAGTCTCCGGCGACGGACGTCGTGACATCGGTG
GTTGTAAAAGACCCTCATGTCTTGTCACGCGAATCTGCGTCATCTTAGTGAGCTCTAGGA
CCCCTAGGCAGCACACACTCTGTGCGCTTCGATTGTGACCCCAGGTCAGGCGGGACTACC
CGCTGAGTTTAAGCATATCAATAAGCGGAGGAAAAGAAACTTACAAGGATTCCCCTAGTA
ACGGCGAGCGAACCGGGAACAGCCCAGCTTGAAAATTGGGCGGCTTTGCCGTCTGAATTG
TAGTCTGAGAAAA-GCGTCA
>6Ligusticum_jeholense_ITS2
TTATGCGATTA-CTGGTGTGAATTGCAGAATCCCGTGAACCATCGAGTCTTTGAACGCAA
GTTGCGCCCGAAGCCATTAGGCTGAGGGCACGTCTGCCTGGGTGTCACGCATCATCTTTG
CCAACAACCACACACTCCTTCAAGAGCTGTGCCGGTTTGGGGCGGAAATTGGCCTCCCGT
GCCTTGTTGTGCGGTTGGCGCAAAAGCGAGTCTCCGGCGACGGACGTCGTGACATCGGTG
GTTGTAAAAGACCCTCATGTCTTGTCACGCGAATCTGCGTCATCTTAGTGAGCTCTAGGA
CCCCTAGGCAGCACACACTCTGTGCGCTTCGATTGTGACCCCAGGTCAGGCGGGACTACC
CGCTGAGTTTAAGCATATCAATAAGCGGAGGAAAAGAAACTTACAAGGATTCCCCTAGTA
ACGGCGAGCGAACCGGGAACAGCCCAGCTTGAAAATTGGGCGGCTTTGCCGTCTGAATTG
TAGTCTGAGGAAA-GCGTCA
>13Ligusticum_jeholense_ITS2
TTATGCGATTA-CTGGTGTGAATTGCAGAATCCCGTGAACCATCGAGTCTTTGAACGCAA
GTTGCGCCCGAAGCCATTAGGCTGAGGGCACGTCTGCCTGGGTGTCACGCATCATCTTTG
CCAACAACCACACACTCCTTCAAGAGCTGTGCCGGTTTGGGGCGGAAATTGGCCTCCCGT
GCCTTGTTGTGCGGTTGGCGCAAAAGCGAGTCTCCGGCGACGGACGTCGTGACATCGGTG
GTTGTAAAAGACCCTCATGTCTTGTCACGCGAATCTGCGTCATCTTAGTGAGCTCTAGGA
CCCCTAGGCAGCACACACTCTGTGCGCTTCGATTGTGACCCCAGGTCAGGCGGGACTACC
CGCTGAGTTTAAGCATATCAATAAGCGGAGGAAAAGAAACTTACAAGGATTCCCCTAGTA
ACGGCGAGCGAACCGGGAACAGCCCAGCTTGAAAATTGGGCGGCTTTGCCGTCTGAATTG
TAGTCTGAGAAAAGCGTCA
>14Ligusticum_jeholense_ITS2
TTATGCGATTA-CTGGTGTGAATTGCAGAATCCCGTGAACCATCGAGTCTTTGAACGCAA
GTTGCGCCCGAAGCCATTAGGCTGAGGGCACGTCTGCCTGGGTGTCACGCATCATCTTTG
CCAACAACCACACACTCCTTCAAGAGCTGTGCCGGTTTGGGGCGGAAATTGGCCTCCCGT
GCCTTGTTGTGCGGTTGGCGCAAAAGCGAGTCTCCGGCGACGGACGTCGTGACATCGGTG
GTTGTAAAAGACCCTCATGTCTTGTCACGCGAATCTGCGTCATCTTAGTGAGCTCTAGGA
CCCCTAGGCAGCACACACTCTGTGCGCTTCGATTGTGACCCCAGGTCAGGCGGGACTACC
CGCTGAGTTTAAGCATATCAATAAGCGGAGGAAAAGAAACTTACAAGGATTCCCCTAGTA
ACGGCGAGCGAACCGGGAACAGCCCAGCTTGAAAATTGGGCGGCTTTGCCGTCTGAATTG
TAGTCTGGGGAAAGCGTCA
>18Ligusticum_jeholense_ITS2
TTATGCGATTA-CTGGTGTGAATTGCAGAATCCCGTGAACCATCGAGTCTTTGAACGCAA
GTTGCGCCCGAAGCCATTAGGCTGAGGGCACGTCTGCCTGGGTGTCACGCATCATCTTTG
CCAACAACCACACACTCCTTCAAGAGCTGTGCCGGTTTGGGGCGGAAATTGGCCTCCCGT
GCCTTGTTGTGCGGTTGGCGCAAAAGCGAGTCTCCGGCGACGGACGTCGTGACATCGGTG
GTTGTAAAAGACCCTCATGTCTTGTCACGCGAATCTGCGTCATCTTAGTGAGCTCTAGGA
CCCCTAGGCAGCACACACTCTGTGCGCTTCGATTGTGACCCCAGGTCAGGCGGGACTACC

CGCTGAGTTTAAGCATATCAATAAGCGGAGGAAAAGAAACTTACAAGGATTCCCCTAGTA
ACGGCGAGCGAACCGGGAACAGCCCAGCTTGAAAATTGGGCGGCTTTGCCGTCTGAATTG
TAGTCTGGGGAAAAGCGTCA
>7Conioselinum_vaginatum_ITS2
TTATGCGATTA-CTGGTGTGAATTGCAGAATCCCGTGAACCATCGAGTCTTTGAACGCAA
GTTGCGCCCGAAGCCACTAGGCTGAGGGCACGTCTGCCTGGGTGTCACGCATCATCTTTG
CCCACAACCACTCACTCCTTGAGGAGCTGTGTCGGTTTGGGGCGGAAATTGGCCTCCCGT
GCCTTGTTGTGCGGTTGGCGCAAAAGCGAGTCTCCGGCGACGGACGTCGTGACATCGGTG
GTTGTAAAAGACCCTCATGTCTTGTCGCGCGAATCCGCGTCATCTTAGTGAGCTCTAGGA
CCCTTAGGCGCCACACACTCTGTGCGCTTCGATTGTGACCCCAGGTCAGGCGGGACTACC
CGCTGAGTTTAAGCATATCAATAAGCGGAGGAAAAGAAACTTACAAGGATTCCCCTAGTA
ACGGCGAGCGAACCGGGAATAGCCCAGCTTGAAAATTGGGCGGCTCTGCCGTCCGAATTG
TAGTCTGAGGAAA-GCGTCA
>8Conioselinum_vaginatum_ITS2
TTATGCGATTA-CTGGTGTGAATTGCAGAATCCCGTGAACCATCGAGTCTTTGAACGCAA
GTTGCGCCCGAAGCCACTAGGCTGAGGGCACGTCTGCCTGGGTGTCACGCATCATCTTTG
CCCACAACCACTCACTCCTTGAGGAGCTGTGTCGGTTTGGGGCGGAAATTGGCCTCCCGT
GCCTTGTTGTGCGGTTGGCGCAAAAGCGAGTCTCCGGCGACGGACGTCGTGACATCGGTG
GTTGTAAAAGACCCTCATGTCTTGTCGCGCGAATCCGCGTCATCTTAGTGAGCTCTAGGA
CCCTTAGGCGCCACACACTCTGTGCGCTTCGATTGTGACCCCAGGTCAGGCGGGACTACC
CGCTGAGTTTAAGCATATCAATAAGCGGAGGAAAAGAAACTTACAAGGATTCCCCTAGTA
ACGGCGAGCGAACCGGGAATAGCCCAGCTTGAAAATTGGGCGGCTCTGCCGTCCGAATTG
TAGTCTGGGGAAA-GCGTCA
>9Conioselinum_vaginatum_ITS2
TTATGCGATTA-CTGGTGTGAATTGCAGAATCCCGTGAACCATCGAGTCTTTGAACGCAA
GTTGCGCCCGAAGCCACTAGGCTGAGGGCACGTCTGCCTGGGTGTCACGCATCATCTTTG
CCCACAACCACTCACTCCTTGAGGAGCTGTGTCGGTTTGGGGCGGAAATTGGCCTCCCGT
GCCTTGTTGTGCGGTTGGCGCAAAAGCGAGTCTCCGGCGACGGACGTCGTGACATCGGTG
GTTGTAAAAGACCCTCATGTCTTGTCGCGCGAATCCGCGTCATCTTAGTGAGCTCTAGGA
CCCTTAGGCGCCACACACTCTGTGCGCTTCGATTGTGACCCCAGGTCAGGCGGGACTACC
CGCTGAGTTTAAGCATATCAATAAGCGGAGGAAAAGAAACTTACAAGGATTCCCCTAGTA
ACGGCGAGCGAACCGGGAATAGCCCAGCTTGAAAATTGGGCGGCTCTGCCGTCCGAATTG
TAGTCTGGGGAAAAGCGTCA
>15Conioselinum_vaginatum_ITS2
TTATGCGATTA-CTGGTGTGAATTGCAGAATCCCGTGAACCATCGAGTCTTTGAACGCAA
GTTGCGCCCGAAGCCACTAGGCTGAGGGCACGTCTGCCTGGGTGTCACGCATCATCTTTG
CCCACAACCACTCACTCCTTGAGGAGCTGTGTCGGTTTGGGGCGGAAATTGGCCTCCCGT
GCCTTGTTGTGCGGTTGGCGCAAAAGCGAGTCTCCGGCGACGGACGTCGTGACATCGGTG
GTTGTAAAAGACCCTCATGTCTTGTCGCGCGAATCCGCGTCATCTTAGTGAGCTCTAGGA
CCCTTAGGCGCCACACACTCTGTGCGCTTCGATTGTGACCCCAGGTCAGGCGGGACTACC
CGCTGAGTTTAAGCATATCAATAAGCGGAGGAAAAGAAACTTACAAGGATTCCCCTAGTA
ACGGCGAGCGAACCGGGAATAGCCCAGCTTGAAAATTGGGCGGCTCTGCCGTCCGAATTG
TAGTCTGGGGAAAAGCGTCA
>16Conioselinum_vaginatum_ITS2
TTATGCGATTA-CTGGTGTGAATTGCAGAATCCCGTGAACCATCGAGTCTTTGAACGCAA
GTTGCGCCCGAAGCCACTAGGCTGAGGGCACGTCTGCCTGGGTGTCACGCATCATCTTTG
CCCACAACCACTCACTCCTTGAGGAGCTGTGTCGGTTTGGGGCGGAAATTGGCCTCCCGT
GCCTTGTTGTGCGGTTGGCGCAAAAGCGAGTCTCCGGCGACGGACGTCGTGACATCGGTG
GTTGTAAAAGACCCTCATGTCTTGTCGCGCGAATCCGCGTCATCTTAGTGAGCTCTAGGA
CCCTTAGGCGCCACACACTCTGTGCGCTTCGATTGTGACCCCAGGTCAGGCGGGACTACC
CGCTGAGTTTAAGCATATCAATAAGCGGAGGAAAAGAAACTTACAAGGATTCCCCTAGTA
ACGGCGAGCGAACCGGGAATAGCCCAGCTTGAAAATTGGGCGGCTCTGCCGTCCGAATTG
TAGTCTGGGGAAAAGCGTCA
>17Conioselinum_vaginatum_ITS2

TTATGCGATTA-CTGGTGTGAATTGCAGAATCCCGTGAACCATCGAGTCTTTGAACGCAA
GTTGCGCCCGAAGCCACTAGGCTGAGGGCACGTCTGCCTGGGTGTCACGCATCATCTTTG
CCCACAACCACTCACTCCTTGAGGAGCTGTGTCGGTTTGGGGCGGAAATTGGCCTCCCGT
GCCTTGTTGTGCGGTTGGCGCAAAAGCGAGTCTCCGGCGACGGACGTCGTGACATCGGTG
GTTGTAAAAGACCCTCATGTCTTGTCGCGCGAATCCGCGTCATCTTAGTGAGCTCTAGGA
CCCTTAGGCGCCACACACTCTGTGCGCTTCGATTGTGACCCCAGGTCAGGCGGGACTACC
CGCTGAGTTTAAGCATATCAATAAGCGGAGGAAAAGAAACTTACAAGGATTCCCCTAGTA
ACGGCGAGCGAACCGGGAATAGCCCAGCTTGAAAATTGGGCGGCTCTGCCGTCCGAATTG
TAGTCTGGGAAAAGCGTCA
