# Supplementary material for: Molecular Structure and Phylogenetic Analyses of the Complete Chloroplast Genomes of Three Medicinal Plants Conioselinum vaginatum, Ligusticum sinense, and Ligusticum jeholense
Source: Front Plant Sci. 2022 Jun 6;13:878263. doi: 10.3389/fpls.2022.878263 (PMC9207526; doi:10.3389/fpls.2022.878263)
Supplement: Supplementary file 3 [file Data_Sheet_3.PDF]

>1Ligusticum_sinense_psbA_trnH
TCCGCCCCGCCAATTTTATTTTATTTATTTTTATTTCAAAGGATTCCTTTTTGATCATTC
AAAAATATTTGTTTATCTAAAACAGTCTGAAATATAAAAAAAAAAGCAATACCGCCCTCTT
GCCTCTTGATAGAACAAGAGGGCGGTATTGCTTTTTTTATTTCAAAAAACTCGTATATAC
TAAAACCCGGTCTTACCCATTTGTAGATGGAGCTTCAACAGCAGCTAGGTCTAGAGGA
>2Ligusticum_sinense_psbA_trnH
TCCGCCCCGCCAATTTTATTTTATTTATTTTTATTTCAAAGGATTCCTTTTTGATCATTC
AAAAATATTTGTTTATCTAAAACAGTCTGAAATATAAAAAAAAAAGCAATACCGCCCTCTT
GCCTCTTGATAGAACAAGAGGGCGGTATTGCTTTTTTTATTTCAAAAAACTCGTATATAC
TAAAACCCGGTCTTACCCATTTGTAGATGGAGCTTCAACAGCAGCTAGGTCTAGAGGA
>3Ligusticum_sinense_psbA_trnH
TCCGCCCCGCCAATTTTCTTTTATTTATTTTTATTTCAAAGGATTCCTTTTTGATCATTC
AAAAATATTTGTTTATCTAAAACAGTCTGAAATATAAAAAAAAAAGCAATACCGCCCTCTT
GCCTCTTGATAGAACAAGAGGGCGGTATTGCTTTTTTTATTTCAAAAAACTCGTATATAC
TAAAACCCGGTCTTACCCATTTGTAGATGGAGCTTCAACAGCAGCTAGGTCTAGAGGA
>11Ligusticum_sinense_psbA_trnH
TCCGCCCCGCCAATTTTATTTTATTTATTTTTATTTCAAAGGATTCCTTTTTGATCATTC
AAAAATATTTGTTTATCTAAAACAGTCTGAAATATAAAAAAAAAAGCAATACCGCCCTCTT
GCCTCTTGATAGAACAAGAGGGCGGTATTGCTTTTTTTATTTCAAAAAACTCGTATATAC
TAAAACCCGGTCTTACCCATTTGTAGATGGAGCTTCAACAGCAGCTAGGTCTAGAGGA
>4Ligusticum_jeholense_psbA_trnH
TCCGCCCCGCCAATTTTATTTTATTTATTTTTATTTCAAAGGATTCCTTTTTGATCATTC
AAAAATATTTGTTTATCTAAAACAGTCTGAAATATAAAAAAAAAAGCAATACCGCCCTCTT
GCCTCTTGATAGAACAAGAGGGCGGTATTGCTTTTTTTATTTCAAAAAACTCGTATATAC
TAAAACCCGGTCTTACCCATTTGTAGATGGAGCTTCAACAGCAGCTAGGTCTAGAGGA
>5Ligusticum_jeholense_psbA_trnH
TCCGCCCCGCCAATTTTATTTTATTTATTTTTATTTCAAAGGATTCCTTTTTGATCATTC
AAAAATATTTGTTTATCTAAAACAGTCTGAAATATAAAAAAAAAAGCAATACCGCCCTCTT
GCCTCTTGATAGAACAAGAGGGCGGTATTGCTTTTTTTATTTCAAAAAACTCGTATATAC
TAAAACCCGGTCTTACCCATTTGTAGATGGAGCTTCAACAGCAGCTAGGTCTAGAGGA
>6Ligusticum_jeholense_psbA_trnH
TCCGCCCCGCCAATTTTATTTTATTTATTTTTATTTCAAAGGATTCCTTTTTGATCATTC
AAAAATATTTGTTTATCTAAAACAGTCTGAAATATAAAAAAAAAAGCAATACCGCCCTCTT
GCCTCTTGATAGAACAAGAGGGCGGTATTGCTTTTTTTATTTCAAAAAACTCGTATATAC
TAAAACCCGGTCTTACCCATTTGTAGATGGAGCTTCAACAGCAGCTAGGTCTAGAGGA
>12Ligusticum_jeholense_psbA_trnH
TCCGCCCCGCCAATTTTATTTTATTTATTTTTATTTCAAAGGATTCCTTTTTGATCATTC
AAAAATATTTGTTTATCTAAAACAGTCTGAAATATAAAAAAAAAAGCAATACCGCCCTCTT
GCCTCTTGATAGAACAAGAGGGCGGTATTGCTTTTTTTATTTCAAAAAACTCGTATATAC
TAAAACCCGGTCTTACCCATTTGTAGATGGAGCTTCAACAGCAGCTAGGTCTAGAGGA
>13Ligusticum_jeholense_psbA_trnH
TCCGCCCCGCCAATTTTATTTTATTTATTTTTATTTCAAAGGATTCCTTTTTGATCATTC
AAAAATATTTGTTTATCTAAAACAGTCTGAAATATAAAAAAAAAAGCAATACCGCCCTCTT
GCCTCTTGATAGAACAAGAGGGCGGTATTGCTTTTTTTATTTCAAAAAACTCGTATATAC
TAAAACCCGGTCTTACCCATTTGTAGATGGAGCTTCAACAGCAGCTAGGTCTAGAGGA
>14Ligusticum_jeholense_psbA_trnH
TCCGCCCCGCCAATTTTATTTTATTTATTTTTATTTCAAAGGATTCCTTTTTGATCATTC
AAAAATATTTGTTTATCTAAAACAGTCTGAAATATAAAAAAAAAAGCAATACCGCCCTCTT
GCCTCTTGATAGAACAAGAGGGCGGTATTGCTTTTTTTATTTCAAAAAACTCGTATATAC
TAAAACCCGGTCTTACCCATTTGTAGATGGAGCTTCAACAGCAGCTAGGTCTAGAGGA
>18Ligusticum_jeholense_psbA_trnH
TCCGCCCCGCCAATTTTATTTTATTTATTTTTATTTCAAAGGATTCCTTTTTGATCATTC
AAAAATATTTGTTTATCTAAAACAGTCTGAAATATAAAAAAAAA-GCAATACCGCCCTCTT

GCCTCTTGATAGAACAAGAGGGCGGTATTGCTTTTTTTATTTCAAAAAACTCGTATATAC
TAAAACCCGGTCTTACCCATTTGTAGATGGAGCTTCAACAGCAGCTAGGTCTAGAGGA
>7Conioselinum_vaginatum_psbA_trnH
TCCGCCCCGCCAATTTTCTTTTATTTATTTTTATTTCAAAGGATTCCTTTTTGATTATTC
AAAAATATTTGTTTATCTAAAACAGTCTGAAATATAAAAAAAA-GCAATACCGCCCTCTT
GTTCTATCAAGAGGCAAGAGGGCGGTATTGCTTTTTTTATTTCAAAAAACTCGTATATAC
TAAAACCCGGTCTTACCCATTTGTAGATGGAGCTTCAACAGCAGCTAGGTCTAGAGGA
>17Conioselinum_vaginatum_psbA_trnH
TCCGCCCCGCCAATTTTCTTTTATTTATTTTTATTTCAAAGGATTCCTTTTTGATTATTC
AAAAATATTTGTTTATCTAAAACAGTCTGAAATATAAAAAAAA-GCAATACCGCCCTCTT
GTTCTATCAAGAGGCAAGAGGGCGGTATTGCTTTTTTTATTTCAAAAAACTCGTATATAC
TAAAACCCGGTCTTACCCATTTGTAGATGGAGCTTCAACAGCAGCTAGGTCTAGAGGA
>8Conioselinum_vaginatum_psbA_trnH
TCCGCCCCGCCAATTTTCTTTTATTTATTTTTATTTCAAAGGATTCCTTTTTGATTATTC
AAAAATATTTGTTTATCTAAAACAGTCTGAAATATAAAAAAAA-GCAATACCGCCCTCTT
GTTCTATCAAGAGGCAAGAGGGCGGTATTGCTTTTTTTATTTCAAAAAACTCGTATATAC
TAAAACCCGGTCTTACCCATTTGTAGATGGAGCTTCAACAGCAGCTAGGTCTAGAGGA
>9Conioselinum_vaginatum_psbA_trnH
TCCGCCCCGCCAATTTTCTTTTATTTATTTTTATTTCAAAGGATTCCTTTTTGATTATTC
AAAAATATTTGTTTATCTAAAACAGTCTGAAATATAAAAAAAA-GCAATACCGCCCTCTT
GTTCTATCAAGAGGCAAGAGGGCGGTATTGCTTTTTTTATTTCAAAAAACTCGTATATAC
TAAAACCCGGTCTTACCCATTTGTAGATGGAGCTTCAACAGCAGCTAGGTCTAGAGGA
>16Conioselinum_vaginatum_psbA_trnH
TCCGCCCCGCCAATTTTATTTTATTTATTTTTATTTCAAAGGATTCCTTTTTGATTATTC
AAAAATATTTGTTTATCTAAAACAGTCTGAAATATAAAAAAAA-GCAATACCGCCCTCTT
GTTCTATCAAGAGGCAAGAGGGCGGTATTGCTTTTTTTATTTCAAAAAACTCGTATATAC
TAAAACCCGGTCTTACCCATTTGTAGATGGAGCTTCAACAGCAGCTAGGTCTAGAGGA
>15Conioselinum_vaginatum_psbA_trnH
TCCGCCCCGCCAATTTTATTTTATTTATTTTTATTTCAAAGGATTCCTTTTTGATTATTC
AAAAATATTTGTTTATCTAAAACAGTCTGAAATATAAAAAAAA-GCAATACCGCCCTCTT
GTTCTATCAAGAGGCAAGAGGGCGGTATTGCTTTTTTTATTTCAAAAAACTCGTATATAC
TAAAACCCGGTCTTACCCATTTGTAGATGGAGCTTCAACAGCAGCTAGGTCTAGAGGA
>10Ligusticum_sp_psbA_trnH
TCCGCCCCGCCAATTTTATTTTATTTATTTTTATTTCAAAGGATTCCTTTTTGATCATTC
AAAAATATTTGTTTATCTAAAACAGTCTGAAATATAAAAAAAA-GCAATACCGCCCTCTT
GTTCTATCAAGAGGCAAGAGGGCGGTATTGCTTTTTTTATTTCAAAAAACTCGTATATAC
TAAAACCCGGTCTTACCCATTTGTAGATGGAGCTTCAACAGCAGCTAGGTCTAGAGGA
