# Supplementary material for: Molecular Structure and Phylogenetic Analyses of the Complete Chloroplast Genomes of Three Medicinal Plants Conioselinum vaginatum, Ligusticum sinense, and Ligusticum jeholense
Source: Front Plant Sci. 2022 Jun 6;13:878263. doi: 10.3389/fpls.2022.878263 (PMC9207526; doi:10.3389/fpls.2022.878263)
Supplement: Supplementary file 4 [file Data_Sheet_4.PDF]

>1Ligusticum_sinense_ycf2_trnL
CGCTTGTTTCTTCCATATTTTGGACCTTAGCTCCCTGGAACAATAGGCTACTGCTGAAAC
ACGGAAGAATTTAAATATTAGATCAAAACACTATGTATGGATGGTATGAACTGCCTAAAC
AAGAATTCTTGAACAGCGAACAACCAGTTCATATATTCACGACCAA--------------

-----------GAAAAGAAGTACTGGATTTTCTTTCGGATAGGCCCTGAAAGCGAAGGAA
GGCTGGAATGCCAACAGGCGTCTATTATTGAATTCACCCAACCCGACAGTACCCATTTTG
GGAACGTCCAGTGCCAAAAGTCACTGAATGGATAAATCGCCAATCCCTAAAACGGACTAT
GTAATGTACTTTATCTGCTGGGTTGCGGGCGGGCATTTTACCAGAGGTTTCTAATCTACC
CTTGTGTGATTCCTGTTGAAGCATATACTCGGGGGGGGGGGGGGTGCAGGGCGGACGATT
TTTAAGCAGACCCCCCATTCATTAGATAGAGAAGATCCCCAAGATTTAGTGATCTGCTGC
CGAAC
>2Ligusticum_sinense_ycf2_trnL
CGCTTGTTTCTTCCATATTTTGGACCTTAGCTCCCTGGAACAATAGGCTACTGCTGAAAC
ACGGAAGAATTTAAATATTAGATCAAAACACTATGTATGGATGGTATGAACTGCCTAAAC
AAGAATTCTTGAACAGCGAACAACCAGTTCATATATTCACGACCAA--------------

-----------GAAAAGAAGTACTGGATTTTCTTTCGGATAGGCCCTGAAAGCGAAGGAA
GGCTGGAATGCCAACAGGCGTCTATTATTGAATTCACCCAACCCGACAGTACCCATTTTG
GGAACGTCCAGTGCCAAAAGTCACTGAATGGATAAATCGCCAATCCCTAAAACGGACTAT
GTAATGTACTTTATCTGCTGGGTTGCGGGCGGGCATTTTACCAGAGGTTTCTAATCTACC
CTTGTGTGATTCCTGTTGAAGCATATACTCGGGGGGGGGGGGGGTGCAGGGCGGACGATT
TTTAAGCAGACCCCCCATTCATTAGATAGAGAAGATCCCCAAGATTTAGTGATCTGCTGC
CGAAC
>3Ligusticum_sinense_ycf2_trnL

CGCTTGTTTCTTCCATATTTTGGACCTTAGCTCCCTGGAACAATAGGCTACTGCTGAAAC
ACGGAAGAATTTAAATATTAGATCAAAACACTATGTATGGATGGTATGAACTGCCTAAAC
AAGAATTCTTGAACAGCGAACAACCAGTTCATATATTCACGACCAA--------------

-----------GAAAAGAAGTACTGGATTTTCTTTCGGATAGGCCCTGAAAGCGAAGGAA
GGCTGGAATGCCAACAGGCGTCTATTATTGAATTCACCCAACCCGACAGTACCCATTTTG
GGAACGTCCAGTGCCAAAAGTCACTGAATGGATAAATCGCCAATCCCTAAAACGGACTAT
GTAATGTACTTTATCTGCTGGGTTGCGGGCGGGCATTTTACCAGAGGTTTCTAATCTACC
CTTGTGTGATTCCTGTTGAAGCATATACTCGGGGGGGGGGG---TGCAGGGCGGACGATT
TTTAAGCAGACCCCCCATTCATTAGATAGAGAAGATCCCCAAGATTTAGTGATCTGCTGC
CGAAC
>11Ligusticum_sinense_ycf2_trnL
CGCTTGTTTCTTCCATATTTTGGACCTTAGCTCCCTGGAACAATAGGCTACTGCTGAAAC
ACGGAAGAATTTAAATATTAGATCAAAACACTATGTATGGATGGTATGAACTGCCTAAAC
AAGAATTCTTGAACAGCGAACAACCAGTTCATATATTCACGACCAA--------------

-----------GAAAAGAAGTACTGGATTTTCTTTCGGATAGGCCCTGAAAGCGAAGGAA
GGCTGGAATGCCAACAGGCGTCTATTATTGAATTCACCCAACCCGACAGTACCCATTTTG
GGAACGTCCAGTGCCAAAAGTCACTGAATGGATAAATCGCCAATCCCTAAAACGGACTAT
GTAATGTACTTTATCTGCTGGGTTGCGGGCGGGCATTTTACCAGAGGTTTCTAATCTACC
CTTGTGTGATTCCTGTTGAAGCATATACTCGGGGGGGGGGGGGGTGCAGGGCGGACGATT
TTTAAGCAGACCCCCCATTCATTAGATAGAGAAGATCCCCAAGATTTAGTGATCTGCTGC
CGAAC
>4Ligusticum_jeholense_ycf2_trnL
CGCTTGTTTCTTCCATATTTTGGACCTTAGCTCCCTGGAACAATAGGCTACTGCTGAAAC
ACGGAAGAATTTAAATATTAGATCAAAACACTATGTATGGATGGTATGAACTGCCTAAAC

AAGAATTCTTGAACAGCGAACAACCGGTTCATATATTCACGACCAA--------------

-----------GAAAAGAAGTACTGGATTTTCTTTCGGATAGGCCCTGAAAGCGAAGGAA
GGCTGGAATGCCAACAGGCGTCTATTATTGAATTCACCCAACCCGACAGTACCCATTTTG
GGAACGTCCAGTGCCAAAAGTCACTGAATGGATAAATCGCCAATCCCTAAAACGGACTAT
GTAATGTACTTTATCTGCTGGGTTGCGGGCGGGCATTTTACCAGAGGTTTCTAATCTACC
CTTGTGTGATTCCTGTTGAAGCATATACTCGGGGGGGGGGGGGGTGCAGGGCGGACGATT
TTTAAGCAGACCCCCCATTCATTAGATAGAGAAGATCCCCAAGATTTAGTGATCTGCTGC
CGAAC
>5Ligusticum_jeholense_ycf2_trnL
CGCTTGTTTCTTCCATATTTTGGACCTTAGCTCCCTGGAACAATAGGCTACTGCTGAAAC
ACGGAAGAATTTAAATATTAGATCAAAACACTATGTATGGATGGTATGAACTGCCTAAAC
AAGAATTCTTGAACAGCGAACAACCGGTTCATATATTCACGACCAA--------------

-----------GAAAAGAAGTACTGGATTTTCTTTCGGATAGGCCCTGAAAGCGAAGGAA
GGCTGGAATGCCAACAGGCGTCTATTATTGAATTCACCCAACCCGACAGTACCCATTTTG
GGAACGTCCAGTGCCAAAAGTCACTGAATGGATAAATCGCCAATCCCTAAAACGGACTAT
GTAATGTACTTTATCTGCTGGGTTGCGGGCGGGCATTTTACCAGAGGTTTCTAATCTACC
CTTGTGTGATTCCTGTTGAAGCATATACTCGGGGGGGGGGGGGGTGCAGGGCGGACGATT
TTTAAGCAGACCCCCCATTCATTAGATAGAGAAGATCCCCAAGATTTAGTGATCTGCTGC
CGAAC
>6Ligusticum_jeholense_ycf2_trnL
CGCTTGTTTCTTCCATATTTTGGACCTTAGCTCCCTGGAACAATAGGCTACTGCTGAAAC
ACGGAAGAATTTAAATATTAGATCAAAACACTATGTATGGATGGTATGAACTGCCTAAAC
AAGAATTCTTGAACAGCGAACAACCGGTTCATATATTCACGACCAA--------------

-----------GAAAAGAAGTACTGGATTTTCTTTCGGATAGGCCCTGAAAGCGAAGGAA
GGCTGGAATGCCAACAGGCGTCTATTATTGAATTCACCCAACCCGACAGTACCCATTTTG
GGAACGTCCAGTGCCAAAAGTCACTGAATGGATAAATCGCCAATCCCTAAAACGGACTAT
GTAATGTACTTTATCTGCTGGGTTGCGGGCGGGCATTTTACCAGAGGTTTCTAATCTACC
CTTGTGTGATTCCTGTTGAAGCATATACTCGGGGGGGGGGGGGGGTGCAGGCGGACGATT
TTTAAGCAGACCCCCCATTCATTAGATAGAGAAGATCCCCAAGATTTAGTGATCTGCTGC
CGAAC
>12Ligusticum_jeholense_ycf2_trnL
CGCTTGTTTCTTCCATATTTTGGACCTTAGCTCCCTGGAACAATAGGCTACTGCTGAAAC
ACGGAAGAATTTAAATATTAGATCAAAACACTATGTATGGATGGTATGAACTGCCTAAAC
AAGAATTCTTGAACAGCGAACAACCGGTTCATATATTCACGACCAA--------------

-----------GAAAAGAAGTACTGGATTTTCTTTCGGATAGGCCCTGAAAGCGAAGGAA
GGCTGGAATGCCAACAGGCGTCTATTATTGAATTCACCCAACCCGACAGTACCCATTTTG
GGAACGTCCAGTGCCAAAAGTCACTGAATGGATAAATCGCCAATCCCTAAAACGGACTAT
GTAATGTACTTTATCTGCTGGGTTGCGGGCGGGCATTTTACCAGAGGTTTCTAATCTACC
CTTGTGTGATTCCTGTTGAAGCATATACTCGGGGGGGGGGGGGG-TGCAGGCGGACGATT
TTTAAGCAGACCCCCCATTCATTAGATAGAGAAGATCCCCAAGATTTAGTGATCTGCTGC
CGAAC
>13Ligusticum_jeholense_ycf2_trnL
CGCTTGTTTCTTCCATATTTTGGACCTTAGCTCCCTGGAACAATAGGCTACTGCTGAAAC
ACGGAAGAATTTAAATATTAGATCAAAACACTATGTATGGATGGTATGAACTGCCTAAAC
AAGAATTCTTGAACAGCGAACAACCGGTTCATATATTCACGACCAA--------------

-----------GAAAAGAAGTACTGGATTTTCTTTCGGATAGGCCCTGAAAGCGAAGGAA
GGCTGGAATGCCAACAGGCGTCTATTATTGAATTCACCCAACCCGACAGTACCCATTTTG
GGAACGTCCAGTGCCAAAAGTCACTGAATGGATAAATCGCCAATCCCTAAAACGGACTAT
GTAATGTACTTTATCTGCTGGGTTGCGGGCGGGCATTTTACCAGAGGTTTCTAATCTACC
CTTGTGTGATTCCTGTTGAAGCATATACTCGGGGGGGGGGGGGGTGCAGGGCGGACGATT
TTTAAGCAGACCCCCCATTCATTAGATAGAGAAGATCCCCAAGATTTAGTGATCTGCTGC
CGAAC
>14Ligusticum_jeholense_ycf2_trnL
CGCTTGTTTCTTCCATATTTTGGACCTTAGCTCCCTGGAACAATAGGCTACTGCTGAAAC
ACGGAAGAATTTAAATATTAGATCAAAACACTATGTATGGATGGTATGAACTGCCTAAAC
AAGAATTCTTGAACAGCGAACAACCGGTTCATATATTCACGACCAA--------------

-----------GAAAAGAAGTACTGGATTTTCTTTCGGATAGGCCCTGAAAGCGAAGGAA
GGCTGGAATGCCAACAGGCGTCTATTATTGAATTCACCCAACCCGACAGTACCCATTTTG
GGAACGTCCAGTGCCAAAAGTCACTGAATGGATAAATCGCCAATCCCTAAAACGGACTAT
GTAATGTACTTTATCTGCTGGGTTGCGGGCGGGCATTTTACCAGAGGTTTCTAATCTACC
CTTGTGTGATTCCTGTTGAAGCATATACTCGGGGGGGGGGGGGGTGCAGGGCGGACGATT
TTTAAGCAGACCCCCCATTCATTAGATAGAGAAGATCCCCAAGATTTAGTGATCTGCTGC
CGAAC
>18Ligusticum_jeholense_ycf2_trnL
CGCTTGTTTCTTCCATATTTTGGACCTTAGCTCCCTGGAACAATAGGCTACTGCTGAAAC
ACGGAAGAATTTAAATATTAGATCAAAACACTATGTATGGATGGTATGAACTGCCTAAAC
AAGAATTCTTGAACAGCGAACAACCGGTTCATATATTCACGACCAA--------------

-----------GAAAAGAAGTACTGGATTTTCTTTCGGATAGGCCCTGAAAGCGAAGGAA
GGCTGGAATGCCAACAGGCGTCTATTATTGAATTCACCCAACCCGACAGTACCCATTTTG
GGAACGTCCAGTGCCAAAAGTCACTGAATGGATAAATCGCCAATCCCTAAAACGGACTAT
GTAATGTACTTTATCTGCTGGGTTGCGGGCGGGCATTTTACCAGAGGTTTCTAATCTACC
CTTGTGTGATTCCTGTTGAAGCATATACTCGGGGGGGGGGGGGGTGCAGGGCGGACGATT
TTTAAGCAGACCCCCCATTCATTAGATAGAGAAGATCCCCAAGATTTAGTGATCTGCTGC
CGAAC
>7Conioselinum_vaginatum_ycf2_trnL
CGCTTGTTTCTTCCATATTTTGGACCTTAGCTCCCTGGAACAAAGGCAGTGGATTGTGAA
TCCACCATGCGCGGGTTCAATTCCCGTCGTTCGCCCGGCGAGCGAGTCTAATCTGATTTC
ACTCCGAAGTTGGTGCAGTTACTAATTCATGATCTGGCATGTACAGCAGGAGGTTTTAGA
TATGTACGATATATCCGTATTCACAGCTCGTCTTCGTTATTCCCATTTTATAAAATCAAT
GGGCGTCTTGGTCTGCCTTTTAGTATTAAATTTTGGATTTCTACCCTTTTTGGGGTTTAA
AATGAAATCCCGTTTCATGAACAACGGGTTTCTTTCGGATCAAGCCCATGCAATGGACTT
TGATCCAACGGAACCCGGAAGGAGCTCACTGGATAGGGTAAGACCTGGTTTTCCCTTATC
CAAAGAGAGCGATATATTGGACGTTTGGGGCGGGATATCTCCACTCAACGACTCCGACTT
ATCCTCATTTGAATCAAATGAGGGCGGAGGTCTGGAAGCAGGACCCTCAAAGGGTCGTTG
TAAAAAAGCTGCCTTACCAAGCAGCAGCCGTGATAAACCCGAAACATTGGCATTAGAAAA
ACCAGTTTTTACATTCAAACCACTGCCATTCCCAAACAAAGAGTTTTATAAACTCAAAAC
AATGGTACTACCAACTAGATCTGATGAACCCATATTAAAACTAGAACCAGGAGATTACCT
GCCATCGGTTTGGAGGGAAAAATTCAACGCAATAAACGAAGCAAGAAACGAAGAGAAATA
CCGACGGTCTGAAATATATACTGTCAGTAAACCCATGTTGGGATCGGAACTGTCGAAAGC
ACATATTAGCAGGATTACACCTAGCTTTAAAATTTTACGCCTGTACACATTACCAATCAG
GGTATCAAACATACACTGGGAGGAAGCAGCGGAAGCCCTCCTGGGGAGGGAAAATGAAGG
CATTTGGAAAGACAAAATAAGGGATCTGCAGCAGCATGGAAAAAATAGTGCTTTCTATAA
ATCCGCGGCTGAATGGTTGATACAAAAAAAAATGGATAGAGGAGGATGTCTTTAATGACA
AAGACATTGTTGAGAAGAAGTACTGGATTTTCTTTCGGATAGGCCCTGAAAGCGAAGGAA
GGCTGGAATGCCAACAGGCGTCTATTATTGAATTCACCCAACCCGACAGTACCCATTTTG
GGAACGTCCAGTGCCAAAAGTCACTGAATGGATAAATCGCCAATCCCTAAAACGGACTAT
GTAATGTACTTTATCTGCTGGGTTGCGGGCGGGCATTTTACCAGAGGTTTCTAATCTACC
CTTGTGTGATTCCTGTTGAAGCATATACTCGGGGGGGGGGGGG-TGCAGGGCGGACGATT
TTTAAGCAGACCCCCCATTCATTAGATAGAGAAGATCCCCAAGATTTAGTGATCTGCTGC
CGAAC
>8Conioselinum_vaginatum_ycf2_trnL
CGCTTGTTTCTTCCATATTTTGGACCTTAGCTCCCTGGAACAAAGGCAGTGGATTGTGAA
TCCACCATGCGCGGGTTCAATTCCCGTCGTTCGCCCGGCGAGCGAGTCTAATCTGATTTC
ACTCCGAAGTTGGTGCAGTTACTAATTCATGATCTGGCATGTACAGCAGGAGGTTTTAGA
TATGTACGATATATCCGTATTCACAGCTCGTCTTCGTTATTCCCATTTTATAAAATCAAT
GGGCGTCTTGGTCTGCCTTTTAGTATTAAATTTTGGATTTCTACCCTTTTTGGGGTTTAA
AATGAAATCCCGTTTCATGAACAACGGGTTTCTTTCGGATCAAGCCCATGCAATGGACTT
TGATCCAACGGAACCCGGAAGGAGCTCACTGGATAGGGTAAGACCTGGTTTTCCCTTATC
CAAAGAGAGCGATATATTGGACGTTTGGGGCGGGATATCTCCACTCAACGACTCCGACTT
ATCCTCATTTGAATCAAATGAGGGCGGAGGTCTGGAAGCAGGACCCTCAAAGGGTCGTTG
TAAAAAAGCTGCCTTACCAAGCAGCAGCCGTGATAAACCCGAAACATTGGCATTAGAAAA

ACCAGTTTTTACATTCAAACCACTGCCATTCCCAAACAAAGAGTTTTATAAACTCAAAAC
AATGGTACTACCAACTAGATCTGATGAACCCATATTAAAACTAGAACCAGGAGATTACCT
GCCATCGGTTTGGAGGGAAAAATTCAACGCAATAAACGAAGCAAGAAACGAAGAGAAATA
CCGACGGTCTGAAATATATACTGTCAGTAAACCCATGTTGGGATCGGAACTGTCGAAAGC
ACATATTAGCAGGATTACACCTAGCTTTAAAATTTTACGCCTGTACACATTACCAATCAG
GGTATCAAACATACACTGGGAGGAAGCAGCGGAAGCCCTCCTGGGGAGGGAAAATGAAGG
CATTTGGAAAGACAAAATAAGGGATCTGCAGCAGCATGGAAAAAATAGTGCTTTCTATAA
ATCCGCGGCTGAATGGTTGATACAAAAAAAAATGGATAGAGGAGGATGTCTTTAATGACA
AAGACATTGTTGAGAAGAAGTACTGGATTTTCTTTCGGATAGGCCCTGAAAGCGAAGGAA
GGCTGGAATGCCAACAGGCGTCTATTATTGAATTCACCCAACCCGACAGTACCCATTTTG
GGAACGTCCAGTGCCAAAAGTCACTGAATGGATAAATCGCCAATCCCTAAAACGGACTAT
GTAATGTACTTTATCTGCTGGGTTGCGGGCGGGCATTTTACCAGAGGTTTCTAATCTACC
CTTGTGTGATTCCTGTTGAAGCATATACTCGGGGGGGGGGGGG-TGCAGGGCGGACGATT
TTTAAGCAGACCCCCCATTCATTAGATAGAGAAGATCCCCAAGATTTAGTGATCTGCTGC
CGAAC
>9Conioselinum_vaginatum_ycf2_trnL
CGCTTGTTTCTTCCATATTTTGGACCTTAGCTCCCTGGAACAAAGGCAGTGGATTGTGAA
TCCACCATGCGCGGGTTCAATTCCCGTCGTTCGCCCGGCGAGCGAGTCTAATCTGATTTC
ACTCCGAAGTTGGTGCAGTTACTAATTCATGATCTGGCATGTACAGCAGGAGGTTTTAGA
TATGTACGATATATCCGTATTCACAGCTCGTCTTCGTTATTCCCATTTTATAAAATCAAT
GGGCGTCTTGGTCTGCCTTTTAGTATTAAATTTTGGATTTCTACCCTTTTTGGGGTTTAA
AATGAAATCCCGTTTCATGAACAACGGGTTTCTTTCGGATCAAGCCCATGCAATGGACTT
TGATCCAACGGAACCCGGAAGGAGCTCACTGGATAGGGTAAGACCTGGTTTTCCCTTATC
CAAAGAGAGCGATATATTGGACGTTTGGGGCGGGATATCTCCACTCAACGACTCCGACTT
ATCCTCATTTGAATCAAATGAGGGCGGAGGTCTGGAAGCAGGACCCTCAAAGGGTCGTTG
TAAAAAAGCTGCCTTACCAAGCAGCAGCCGTGATAAACCCGAAACATTGGCATTAGAAAA
ACCAGTTTTTACATTCAAACCACTGCCATTCCCAAACAAAGAGTTTTATAAACTCAAAAC
AATGGTACTACCAACTAGATCTGATGAACCCATATTAAAACTAGAACCAGGAGATTACCT
GCCATCGGTTTGGAGGGAAAAATTCAACGCAATAAACGAAGCAAGAAACGAAGAGAAATA
CCGACGGTCTGAAATATATACTGTCAGTAAACCCATGTTGGGATCGGAACTGTCGAAAGC
ACATATTAGCAGGATTACACCTAGCTTTAAAATTTTACGCCTGTACACATTACCAATCAG
GGTATCAAACATACACTGGGAGGAAGCAGCGGAAGCCCTCCTGGGGAGGGAAAATGAAGG
CATTTGGAAAGACAAAATAAGGGATCTGCAGCAGCATGGAAAAAATAGTGCTTTCTATAA
ATCCGCGGCTGAATGGTTGATACAAAAAAAAATGGATAGAGGAGGATGTCTTTAATGACA
AAGACATTGTTGAGAAGAAGTACTGGATTTTCTTTCGGATAGGCCCTGAAAGCGAAGGAA
GGCTGGAATGCCAACAGGCGTCTATTATTGAATTCACCCAACCCGACAGTACCCATTTTG
GGAACGTCCAGTGCCAAAAGTCACTGAATGGATAAATCGCCAATCCCTAAAACGGACTAT
GTAATGTACTTTATCTGCTGGGTTGCGGGCGGGCATTTTACCAGAGGTTTCTAATCTACC
CTTGTGTGATTCCTGTTGAAGCATATACTCGGGGGGGGGGGGG-TGCAGGGCGGACGATT
TTTAAGCAGACCCCCCATTCATTAGATAGAGAAGATCCCCAAGATTTAGTGATCTGCTGC
CGAAC
>15Conioselinum_vaginatum_ycf2_trnL
CGCTTGTTTCTTCCATATTTTGGACCTTAGCTCCCTGGAACAAAGGCAGTGGATTGTGAA
TCCACCATGCGCGGGTTCAATTCCCGTCGTTCGCCCGGCGAGCGAGTCTAATCTGATTTC
ACTCCGAAGTTGGTGCAGTTACTAATTCATGATCTGGCATGTACAGCAGGAGGTTTTAGA
TATGTACGATATATCCGTATTCACAGCTCGTCTTCGTTATTCCCATTTTATAAAATCAAT
GGGCGTCTTGGTCTGCCTTTTAGTATTAAATTTTGGATTTCTACCCTTTTTGGGGTTTAA
AATGAAATCCCGTTTCATGAACAACGGGTTTCTTTCGGATCAAGCCCATGCAATGGACTT
TGATCCAACGGAACCCGGAAGGAGCTCACTGGATAGGGTAAGACCTGGTTTTCCCTTATC
CAAAGAGAGCGATATATTGGACGTTTGGGGCGGGATATCTCCACTCAACGACTCCGACTT
ATCCTCATTTGAATCAAATGAGGGCGGAGGTCTGGAAGCAGGACCCTCAAAGGGTCGTTG
TAAAAAAGCTGCCTTACCAAGCAGCAGCCGTGATAAACCCGAAACATTGGCATTAGAAAA
ACCAGTTTTTACATTCAAACCACTGCCATTCCCAAACAAAGAGTTTTATAAACTCAAAAC
AATGGTACTACCAACTAGATCTGATGAACCCATATTAAAACTAGAACCAGGAGATTACCT

GCCATCGGTTTGGAGGGAAAAATTCAACGCAATAAACGAAGCAAGAAACGAAGAGAAATA
CCGACGGTCTGAAATATATACTGTCAGTAAACCCATGTTGGGATCGGAACTGTCGAAAGC
ACATATTAGCAGGATTACACCTAGCTTTAAAATTTTACGCCTGTACACATTACCAATCAG
GGTATCAAACATACACTGGGAGGAAGCAGCGGAAGCCCTCCTGGGGAGGGAAAATGAAGG
CATTTGGAAAGACAAAATAAGGGATCTGCAGCAGCATGGAAAAAATAGTGCTTTCTATAA
ATCCGCGGCTGAATGGTTGATACAAAAAAAAATGGATAGAGGAGGATGTCTTTAATGACA
AAGACATTGTTGAGAAGAAGTACTGGATTTTCTTTCGGATAGGCCCTGAAAGCGAAGGAA
GGCTGGAATGCCAACAGGCGTCTATTATTGAATTCACCCAACCCGACAGTACCCATTTTG
GGAACGTCCAGTGCCAAAAGTCACTGAATGGATAAATCGCCAATCCCTAAAACGGACTAT
GTAATGTACTTTATCTGCTGGGTTGCGGGCGGGCATTTTACCAGAGGTTTCTAATCTACC
CTTGTGTGATTCCTGTTGAAGCATATACTCGGGGGGGGGGGGG-TGCAGGGCGGACGATT
TTTAAGCAGACCCCCCATTCATTAGATAGAGAAGATCCCCAAGATTTAGTGATCTGCTGC
CGAAC
>16Conioselinum_vaginatum_ycf2_trnL
CGCTTGTTTCTTCCATATTTTGGACCTTAGCTCCCTGGAACAAAGGCAGTGGATTGTGAA
TCCACCATGCGCGGGTTCAATTCCCGTCGTTCGCCCGGCGAGCGAGTCTAATCTGATTTC
ACTCCGAAGTTGGTGCAGTTACTAATTCATGATCTGGCATGTACAGCAGGAGGTTTTAGA
TATGTACGATATATCCGTATTCACAGCTCGTCTTCGTTATTCCCATTTTATAAAATCAAT
GGGCGTCTTGGTCTGCCTTTTAGTATTAAATTTTGGATTTCTACCCTTTTTGGGGTTTAA
AATGAAATCCCGTTTCATGAACAACGGGTTTCTTTCGGATCAAGCCCATGCAATGGACTT
TGATCCAACGGAACCCGGAAGGAGCTCACTGGATAGGGTAAGACCTGGTTTTCCCTTATC
CAAAGAGAGCGATATATTGGACGTTTGGGGCGGGATATCTCCACTCAACGACTCCGACTT
ATCCTCATTTGAATCAAATGAGGGCGGAGGTCTGGAAGCAGGACCCTCAAAGGGTCGTTG
TAAAAAAGCTGCCTTACCAAGCAGCAGCCGTGATAAACCCGAAACATTGGCATTAGAAAA
ACCAGTTTTTACATTCAAACCACTGCCATTCCCAAACAAAGAGTTTTATAAACTCAAAAC
AATGGTACTACCAACTAGATCTGATGAACCCATATTAAAACTAGAACCAGGAGATTACCT
GCCATCGGTTTGGAGGGAAAAATTCAACGCAATAAACGAAGCAAGAAACGAAGAGAAATA
CCGACGGTCTGAAATATATACTGTCAGTAAACCCATGTTGGGATCGGAACTGTCGAAAGC
ACATATTAGCAGGATTACACCTAGCTTTAAAATTTTACGCCTGTACACATTACCAATCAG
GGTATCAAACATACACTGGGAGGAAGCAGCGGAAGCCCTCCTGGGGAGGGAAAATGAAGG
CATTTGGAAAGACAAAATAAGGGATCTGCAGCAGCATGGAAAAAATAGTGCTTTCTATAA
ATCCGCGGCTGAATGGTTGATACAAAAAAAAATGGATAGAGGAGGATGTCTTTAATGACA
AAGACATTGTTGAGAAGAAGTACTGGATTTTCTTTCGGATAGGCCCTGAAAGCGAAGGAA
GGCTGGAATGCCAACAGGCGTCTATTATTGAATTCACCCAACCCGACAGTACCCATTTTG
GGAACGTCCAGTGCCAAAAGTCACTGAATGGATAAATCGCCAATCCCTAAAACGGACTAT
GTAATGTACTTTATCTGCTGGGTTGCGGGCGGGCATTTTACCAGAGGTTTCTAATCTACC
CTTGTGTGATTCCTGTTGAAGCATATACTCGGGGGGGGGGGGGG-TGCAGGGCGGACGATT
TTTAAGCAGACCCCCCATTCATTAGATAGAGAAGATCCCCAAGATTTAGTGATCTGCTGC
CGAAC
>17Conioselinum_vaginatum_ycf2_trnL
CGCTTGTTTCTTCCATATTTTGGACCTTAGCTCCCTGGAACAAAGGCAGTGGATTGTGAA
TCCACCATGCGCGGGTTCAATTCCCGTCGTTCGCCCGGCGAGCGAGTCTAATCTGATTTC
ACTCCGAAGTTGGTGCAGTTACTAATTCATGATCTGGCATGTACAGCAGGAGGTTTTAGA
TATGTACGATATATCCGTATTCACAGCTCGTCTTCGTTATTCCCATTTTATAAAATCAAT
GGGCGTCTTGGTCTGCCTTTTAGTATTAAATTTTGGATTTCTACCCTTTTTGGGGTTTAA
AATGAAATCCCGTTTCATGAACAACGGGTTTCTTTCGGATCAAGCCCATGCAATGGACTT
TGATCCAACGGAACCCGGAAGGAGCTCACTGGATAGGGTAAGACCTGGTTTTCCCTTATC
CAAAGAGAGCGATATATTGGACGTTTGGGGCGGGATATCTCCACTCAACGACTCCGACTT
ATCCTCATTTGAATCAAATGAGGGCGGAKGTCTGGAAGCAKGACCCTCAAAGGGTCGTTG
TAAAAAAGCTGCCTTACCAAGCAGCAGCCGTGATAAACCCGAAACATTGGCATTAGAAAA
ACCAGTTTTTACATTCAAACCACTGCCATTCCCAAACAAAGAGTTTTATAAACTCAAAAC
AATGGTACTACCAACTAGATCTGATGAACCCATATTAAAACTAGAACCAGGAGATTACCT
GCCATCGGTTTGGAGGGAAAAATTCAACGCAATAAACGAAGCAAGAAACGAAGAGAAATA
CCGACGGTCTGAAATATATACTGTCAGTAAACCCATGTTGGGATCGGAACTGTCGAAAGC

ACATATTAGCAGGATTACACCTAGCTTTAAAATTTTACGCCTGTACACATTACCAATCAG
GGTATCAAACATACACTGGGAGGAAGCAGCGGAAGCCCTCCTGGGGAGGGAAAATGAAGG
CATTTGGAAAGACAAAATAAGGGATCTGCAGCAGCATGGAAAAAAATAGTGCTTTCTATAA
ATCCGCGGCTGAATGGTTGATACAAAAAAAAAATGGATAGAGGAGGATGTCTTTAATGACA
AAGACATTGTTGAGAAGAAGTACTGGATTTTCTTTCGGATAGGCCCTGAAAGCGAAGGAA
GGCTGGAATGCCAACAGGCGTCTATTATTGAATTCACCCAACCCGACAGTACCCATTTTG
GGAACGTCCAGTGCCAAAAGTCACTGAATGGATAAATCGCCAATCCCTAAAACGGACTAT
GTAATGTACTTTATCTGCTGGGTTGCGGGCGGGCATTTTACCAGAGGTTTCTAATCTACC
CTTGTGTGATTCCTGTTGAAGCATATACTCGGGGGGGGGGGGGG-TGCAGGGCGGACGATT
TTTAAGCAGACCCCCCATTCATTAGATAGAGAAGATCCCCAAGATTTAGTGATCTGCTGC
CGAAC
