# Supplementary material for: Molecular Structure and Phylogenetic Analyses of the Complete Chloroplast Genomes of Three Medicinal Plants Conioselinum vaginatum, Ligusticum sinense, and Ligusticum jeholense
Source: Front Plant Sci. 2022 Jun 6;13:878263. doi: 10.3389/fpls.2022.878263 (PMC9207526; doi:10.3389/fpls.2022.878263)
Supplement: Supplementary file 5 [file Data_Sheet_5.docx]

Table S1 Primers sequence and amplification procedure

| ID | Primer Name | Primer sequence (5'to3') | Amplification procedure |
| --- | --- | --- | --- |
| *ycf*2-*trn*L | 1F | AAGAGGGGGATTAAGTGGAACA | 94℃3 min 94℃30s, 50℃30s, 72℃1.5 min, 35 cycles 72℃5 min |
|  | 1R | ATATTGAGAATGCTCATTGAATGG |  |
| *acc*D-*ycf*4 | 2F | AAGAGGCCTCTATCAACAAGAA | 94℃5 min 94℃1 min, 55℃1 min, 72℃1.5 min, 30 cycles 72℃7 min |
|  | 2R | AAGTGAGTATAGAAGGTACAAAAGCA |  |
| *psb*A-*trn*H | psbA1F | CGCGCATGGTGGATTCACAATCC | 94℃5 min 94℃1 min, 55℃1 min, 72℃1.5 min, 30 cycles 72℃7 min |
|  | trnH1R | GTTATGCATGAACGTAATGCTC |  |
| ITS2 | IT2L | ATGCGATACTTGGTGTGAAT | 94℃5 min， 94℃30 s, 56℃30 s, 72℃45 s, 40 cycles 72℃10 min |
|  | IT2R | GACGCTTCTCCAGACTACAAT |  |

Table S2 Voucher information of all plant materials sampled of this study.

| No. | Voucher specimen No. | Species name | Collection locality | Plant tissue |
| --- | --- | --- | --- | --- |
| 1 | TCMCQ1 | *Ligusticum sinense* | Chongqing, China | root, medicinal slices |
| 2 | TCMCQ2 | *Ligusticum sinense* | Chongqing, China | root, medicinal slices |
| 3 | TCMCQ3 | *Ligusticum sinense* | Chongqing, China | root, medicinal slices |
| 4 | TCMLN1 | *Ligusticum jeholense* | Liaoning，China | root, medicinal slices |
| 5 | TCMLN2 | *Ligusticum jeholense* | Liaoning，China | root, medicinal slices |
| 6 | TCMLN3 | *Ligusticum jeholense* | Liaoning，China | root, medicinal slices |
| 7 | TCMXJ1 | *Conioselinum vaginatum* | Xinjiang, China | root, medicinal slices |
| 8 | TCMXJ2 | *Conioselinum vaginatum* | Xinjiang, China | root, medicinal slices |
| 9 | TCMXJ3 | *Conioselinum vaginatum* | Xinjiang, China | root, medicinal slices |
| 10 | TCMSC1 | *Ligusticum* sp. | Sichuan，China | root, medicinal slices |
| 11 | 2017072501 | *Ligusticum sinense* | Chengkou County, Chongqing, China | leaf |
| 12 | 2017090101 | *Ligusticum jeholense* | Jiaohe County, Jilin, China | leaf |
| 13 | 2021LGB01 | *Ligusticum jeholense* | Liuhe County, Tonghua City, Jilin, China | leaf |
| 14 | 2021LGB02 | *Ligusticum jeholense* | Qingyuan Manchu Autonomous County,Fushun City,Liaoning, China | leaf |
| 15 | 2018080504 | *Conioselinum vaginatum* | Nilka County,Yining City, Xijiang，China | leaf |
| 16 | 2018080506 | *Conioselinum vaginatum* | Nilka County,Yining City,Xijiang，China | leaf |
| 17 | 2017072522 | *Conioselinum vaginatum* | Nilka County,Yining City,Xijiang，China | leaf |
| 18 | 2017091301 | *Ligusticum jeholense* | Yingermen Town, Qingyuan Manchu Autonomous County,Fushun City,Liaoning, China | leaf |

Table S3 Genbank accession number of species used in the phylogenetic analyses.

| Species name | Genbank accession number |
| --- | --- |
| *Anethum graveolens* L. | KR011055 |
| *Angelica acutiloba* (Sieb. et Zucc.) Kitagawa | KT963036 |
| *Angelica dahurica* (Fisch. ex Hoffm.) Benth. et Hook. f. ex Franch. et | KT963037 |
| *Angelica gigas* Nakai | KT963038 |
| *Anthriscus cerefolium* (L.) Hoffm. | GU456628 |
| *Apium graveolens* L*.* | MK036045 |
| *Arracacia xanthorrhiza* B. | KY117235 |
| *Bupleurum boissieuanum* H. Wolff | MF663725 |
| *Bupleurum latissimum* Nakai | KT983258 |
| *Carum carvi* L | KR048286 |
| *Cicuta virosa* L. | KX352466 |
| *Conioselinum vaginatum* (Spreng.) Thell. | OM728581 (this study) |
| *Coriandrum sativum* L*.* | KR002656 |
| *Crithmum maritimum* L*.* | HM596072 |
| *Daucus carota subsp. sativus* (Hoffm.) Schübl. & G. Martens | DQ898156 |
| *Foeniculum vulgare* Mill. | KR011054 |
| *Glehnia littoralis* Fr. Schmidt ex Miq. | KU866532 |
| *Hansenia forbesii* (H. Boissieu) Pimenov & Kljuykov | KX808492 |
| *Hansenia forrestii* Hansenia forrestii (H. Wolff) Pimenov & Kljuykov | KX808494 |
| *Hansenia oviformis* (R. H. Shan) Pimenov & Kljuykov | KX808493 |
| *Hansenia weberbaueriana* (Fedde ex H. Wolff) Pimenov & Kljuykov | KX808491 |
| *Ledebouriella seseloides* (Hoffm.) H. Wolff | KU866529 |
| *Ligusticum jeholense* Nakai et Kitag | OM728580 (this study) |
| *Ligusticum jeholense* Nakai et Kitag | MN652885 |
| *Ligusticum sinense* Oliv. | OM728579 (this study) |
| *Ligusticum sinense* Oliv. | NC038088 |
| *Ligusticum tenuissimum* (Nakai) Kitag. | KT963039 |
| *Ostericum grosseserratum* (Maxim.) Kitagawa | KT852844 |
| *Panax notoginseng* (Burkill) F. H. Chen ex C. Chow & W. G. Huang | KJ566590 |
| *Pastinaca pimpinellifolia* M. Bieb. | KM035850 |
| *Petroselinum crispum* (Mill.) Hill. | HM596073 |
| *Peucedanum insolens* Kitag. | KU041143 |
| *Peucedanum japonicum* Thunb. | KU866531 |
| *Pleurospermum camtschaticum* Hoffm. | KU041142 |
| *Prangos trifida* (Mill.) Herrnst. & Heyn | MG386251 |
| *Pterygopleurum neurophyllum* (Maxim.) Kitag. | KT983257 |
| *Seseli montanum* L. | KM035851 |

Table S4 Codon usage frequency and RSCU value of the *Conioselinum vaginatum*, *Ligusticum sinense* and *Ligusticum jeholen* chloroplast genomes. RSCU = relative synonymous codon usage.

| *C. vaginatum* | | | | | | | | | |
| --- | --- | --- | --- | --- | --- | --- | --- | --- | --- |
| Amino acid | Codon | No. | RSCU | tRNA | Amino acid | Codon | No. | RSCU | tRNA |
| Phe | UUU | 934 | 1.34 |  | Tyr | UAU | 737 | 1.61 |  |
| Phe | UUC | 460 | 0.66 | *trnF-GAA* | Tyr | UAC | 181 | 0.39 | *trnY-GUA* |
| Leu | UUA | 833 | 1.94 | *trnL-UAA* | Stop | UAA | 46 | 1.62 |  |
| Leu | UUG | 500 | 1.17 | *trnL-CAA* | Stop | UAG | 22 | 0.78 |  |
| Leu | CUU | 534 | 1.25 |  | His | CAU | 426 | 1.5 |  |
| Leu | CUC | 185 | 0.43 |  | His | CAC | 143 | 0.5 | *trnH-GUG* |
| Leu | CUA | 362 | 0.84 | *trnL-UAG* | Gln | CAA | 646 | 1.52 | *trnQ-UUG* |
| Leu | CUG | 159 | 0.37 |  | Gln | CAG | 202 | 0.48 |  |
| Ile | AUU | 996 | 1.44 |  | Asn | AAU | 851 | 1.5 |  |
| Ile | AUC | 394 | 0.57 | *trnI-GAU* | Asn | AAC | 281 | 0.5 | *trnN-GUU* |
| Ile | AUA | 682 | 0.99 | *trnI-CAU* | Lys | AAA | 981 | 1.51 | *trnK-UUU* |
| Met | AUG | 576 | 1 | *trn(f)M-CAU* | Lys | AAG | 319 | 0.49 |  |
| Val | GUU | 515 | 1.5 |  | Asp | GAU | 757 | 1.6 |  |
| Val | GUC | 162 | 0.47 | *trnV-GAC* | Asp | GAC | 187 | 0.4 | *trnD-GUC* |
| Val | GUA | 499 | 1.46 | *trnV-UAC* | Glu | GAA | 937 | 1.51 | *trnE-UUC* |
| Val | GUG | 193 | 0.56 |  | Glu | GAG | 303 | 0.49 |  |
| Ser | UCU | 528 | 1.73 |  | Cys | UGU | 197 | 1.5 |  |
| Ser | UCC | 285 | 0.94 | *trnS-GGA* | Cys | UGC | 65 | 0.5 | *trnC-GCA* |
| Ser | UCA | 352 | 1.16 | *trnS-UGA* | Stop | UGA | 17 | 0.6 |  |
| Ser | UCG | 185 | 0.61 |  | Trp | UGG | 419 | 1 | *trnW-CCA* |
| Pro | CCU | 405 | 1.59 |  | Arg | CGU | 325 | 1.35 | *trnR-ACG* |
| Pro | CCC | 183 | 0.72 |  | Arg | CGC | 92 | 0.38 |  |
| Pro | CCA | 280 | 1.1 | *trnP-UGG* | Arg | CGA | 339 | 1.4 |  |
| Pro | CCG | 153 | 0.6 |  | Arg | CGG | 116 | 0.48 |  |
| Thr | ACU | 526 | 1.64 |  | Arg | AGA | 366 | 1.2 | *trnR-UCU* |
| Thr | ACC | 239 | 0.75 | *trnT-GGU* | Arg | AGG | 110 | 0.36 |  |
| Thr | ACA | 378 | 1.18 | *trnT-UGU* | Ser | AGU | 444 | 1.84 |  |
| Thr | ACG | 140 | 0.44 |  | Ser | AGC | 133 | 0.55 | *trnS-GCU* |
| Ala | GCU | 601 | 1.78 |  | Gly | GGU | 563 | 1.33 |  |
| Ala | GCC | 218 | 0.65 |  | Gly | GGC | 187 | 0.44 | *trnG-GCC* |
| Ala | GCA | 370 | 1.1 | *trnA-UGC* | Gly | GGA | 660 | 1.56 | *trnG-UCC* |
| Ala | GCG | 160 | 0.47 |  | Gly | GGG | 281 | 0.66 |  |
|  |  |  |  |  |  |  |  |  |  |
| *L. jeholense* | | | | | | | | | |
| Amino acid | Codon | No. | RSCU | tRNA | Amino acid | Codon | No. | RSCU | tRNA |
| Phe | UUU | 937 | 1.34 |  | Tyr | UAU | 734 | 1.6 |  |
| Phe | UUC | 457 | 0.66 | *trnF-GAA* | Tyr | UAC | 181 | 0.4 | *trnY-GUA* |
| Leu | UUA | 835 | 1.94 | *trnL-UAA* | Stop | UAA | 45 | 1.59 |  |
| Leu | UUG | 498 | 1.16 | *trnL-CAA* | Stop | UAG | 22 | 0.78 |  |
| Leu | CUU | 535 | 1.25 |  | His | CAU | 427 | 1.5 |  |
| Leu | CUC | 186 | 0.43 |  | His | CAC | 143 | 0.5 | *trnH-GUG* |
| Leu | CUA | 366 | 0.85 | *trnL-UAG* | Gln | CAA | 644 | 1.52 | *trnQ-UUG* |
| Leu | CUG | 158 | 0.37 |  | Gln | CAG | 205 | 0.48 |  |
| Ile | AUU | 996 | 1.44 |  | Asn | AAU | 857 | 1.51 |  |
| Ile | AUC | 395 | 0.57 | *trnI-GAU* | Asn | AAC | 278 | 0.49 | *trnN-GUU* |
| Ile | AUA | 685 | 0.99 | *trnI-CAU* | Lys | AAA | 984 | 1.51 | *trnK-UUU* |
| Met | AUG | 577 | 1 | *trn(f)M-CAU* | Lys | AAG | 323 | 0.49 |  |
| Val | GUU | 513 | 1.5 |  | Asp | GAU | 758 | 1.6 |  |
| Val | GUC | 161 | 0.47 | *trnV-GAC* | Asp | GAC | 189 | 0.4 | *trnD-GUC* |
| Val | GUA | 500 | 1.46 | *trnV-UAC* | Glu | GAA | 934 | 1.51 | *trnE-UUC* |
| Val | GUG | 195 | 0.57 |  | Glu | GAG | 306 | 0.49 |  |
| Ser | UCU | 527 | 1.73 |  | Cys | UGU | 198 | 1.5 |  |
| Ser | UCC | 284 | 0.93 | *trnS-GGA* | Cys | UGC | 66 | 0.5 | *trnC-GCA* |
| Ser | UCA | 350 | 1.15 | *trnS-UGA* | Stop | UGA | 18 | 0.64 |  |
| Ser | UCG | 187 | 0.62 |  | Trp | UGG | 420 | 1 | *trnW-CCA* |
| Pro | CCU | 403 | 1.58 |  | Arg | CGU | 326 | 1.35 | *trnR-ACG* |
| Pro | CCC | 184 | 0.72 |  | Arg | CGC | 91 | 0.38 |  |
| Pro | CCA | 279 | 1.09 | *trnP-UGG* | Arg | CGA | 335 | 1.39 |  |
| Pro | CCG | 154 | 0.6 |  | Arg | CGG | 117 | 0.48 |  |
| Thr | ACU | 528 | 1.64 |  | Arg | AGA | 366 | 1.2 | *trnR-UCU* |
| Thr | ACC | 237 | 0.74 | *trnT-GGU* | Arg | AGG | 110 | 0.36 |  |
| Thr | ACA | 378 | 1.17 | *trnT-UGU* | Ser | AGU | 447 | 1.85 |  |
| Thr | ACG | 144 | 0.45 |  | Ser | AGC | 133 | 0.55 | *trnS-GCU* |
| Ala | GCU | 603 | 1.79 |  | Gly | GGU | 563 | 1.33 |  |
| Ala | GCC | 217 | 0.65 |  | Gly | GGC | 187 | 0.44 | *trnG-GCC* |
| Ala | GCA | 369 | 1.1 | *trnA-UGC* | Gly | GGA | 663 | 1.57 | *trnG-UCC* |
| Ala | GCG | 156 | 0.46 |  | Gly | GGG | 279 | 0.66 |  |
|  |  |  |  |  |  |  |  |  |  |
| *L. sinense* | | | | | | | | | |
| Amino acid | Codon | No. | RSCU | tRNA | Amino acid | Codon | No. | RSCU | tRNA |
| Phe | UUU | 937 | 1.34 |  | Tyr | UAU | 734 | 1.6 |  |
| Phe | UUC | 460 | 0.66 | *trnF-GAA* | Tyr | UAC | 181 | 0.4 | *trnY-GUA* |
| Leu | UUA | 838 | 1.95 | *trnL-UAA* | Stop | UAA | 45 | 1.59 |  |
| Leu | UUG | 497 | 1.15 | *trnL-CAA* | Stop | UAG | 22 | 0.78 |  |
| Leu | CUU | 537 | 1.25 |  | His | CAU | 427 | 1.5 |  |
| Leu | CUC | 186 | 0.43 |  | His | CAC | 143 | 0.5 | *trnH-GUG* |
| Leu | CUA | 366 | 0.85 | *trnL-UAG* | Gln | CAA | 644 | 1.52 | *trnQ-UUG* |
| Leu | CUG | 158 | 0.37 |  | Gln | CAG | 205 | 0.48 |  |
| Ile | AUU | 998 | 1.44 |  | Asn | AAU | 862 | 1.51 |  |
| Ile | AUC | 398 | 0.57 | *trnI-GAU* | Asn | AAC | 279 | 0.49 | *trnN-GUU* |
| Ile | AUA | 685 | 0.99 | *trnI-CAU* | Lys | AAA | 983 | 1.5 | *trnK-UUU* |
| Met | AUG | 577 | 1 | *trn(f)M-CAU* | Lys | AAG | 328 | 0.5 |  |
| Val | GUU | 515 | 1.5 |  | Asp | GAU | 763 | 1.6 |  |
| Val | GUC | 161 | 0.47 | *trnV-GAC* | Asp | GAC | 190 | 0.4 | *trnD-GUC* |
| Val | GUA | 499 | 1.46 | *trnV-UAC* | Glu | GAA | 936 | 1.51 | *trnE-UUC* |
| Val | GUG | 195 | 0.57 |  | Glu | GAG | 306 | 0.49 |  |
| Ser | UCU | 528 | 1.73 |  | Cys | UGU | 198 | 1.5 |  |
| Ser | UCC | 286 | 0.94 | *trnS-GGA* | Cys | UGC | 66 | 0.5 | *trnC-GCA* |
| Ser | UCA | 354 | 1.16 | *trnS-UGA* | Stop | UGA | 18 | 0.64 |  |
| Ser | UCG | 186 | 0.61 |  | Trp | UGG | 424 | 1 | *trnW-CCA* |
| Pro | CCU | 404 | 1.58 |  | Arg | CGU | 326 | 1.35 | *trnR-ACG* |
| Pro | CCC | 184 | 0.72 |  | Arg | CGC | 91 | 0.38 |  |
| Pro | CCA | 279 | 1.09 | *trnP-UGG* | Arg | CGA | 334 | 1.38 |  |
| Pro | CCG | 154 | 0.6 |  | Arg | CGG | 117 | 0.49 |  |
| Thr | ACU | 528 | 1.64 |  | Arg | AGA | 368 | 1.21 | *trnR-UCU* |
| Thr | ACC | 238 | 0.74 | *trnT-GGU* | Arg | AGG | 110 | 0.36 |  |
| Thr | ACA | 378 | 1.17 | *trnT-UGU* | Ser | AGU | 447 | 1.85 |  |
| Thr | ACG | 144 | 0.45 |  | Ser | AGC | 132 | 0.55 | *trnS-GCU* |
| Ala | GCU | 605 | 1.8 |  | Gly | GGU | 564 | 1.33 |  |
| Ala | GCC | 215 | 0.64 |  | Gly | GGC | 187 | 0.44 | *trnG-GCC* |
| Ala | GCA | 369 | 1.1 | *trnA-UGC* | Gly | GGA | 666 | 1.57 | *trnG-UCC* |
| Ala | GCG | 157 | 0.47 |  | Gly | GGG | 280 | 0.66 |  |
|  | | | | | | | | | |


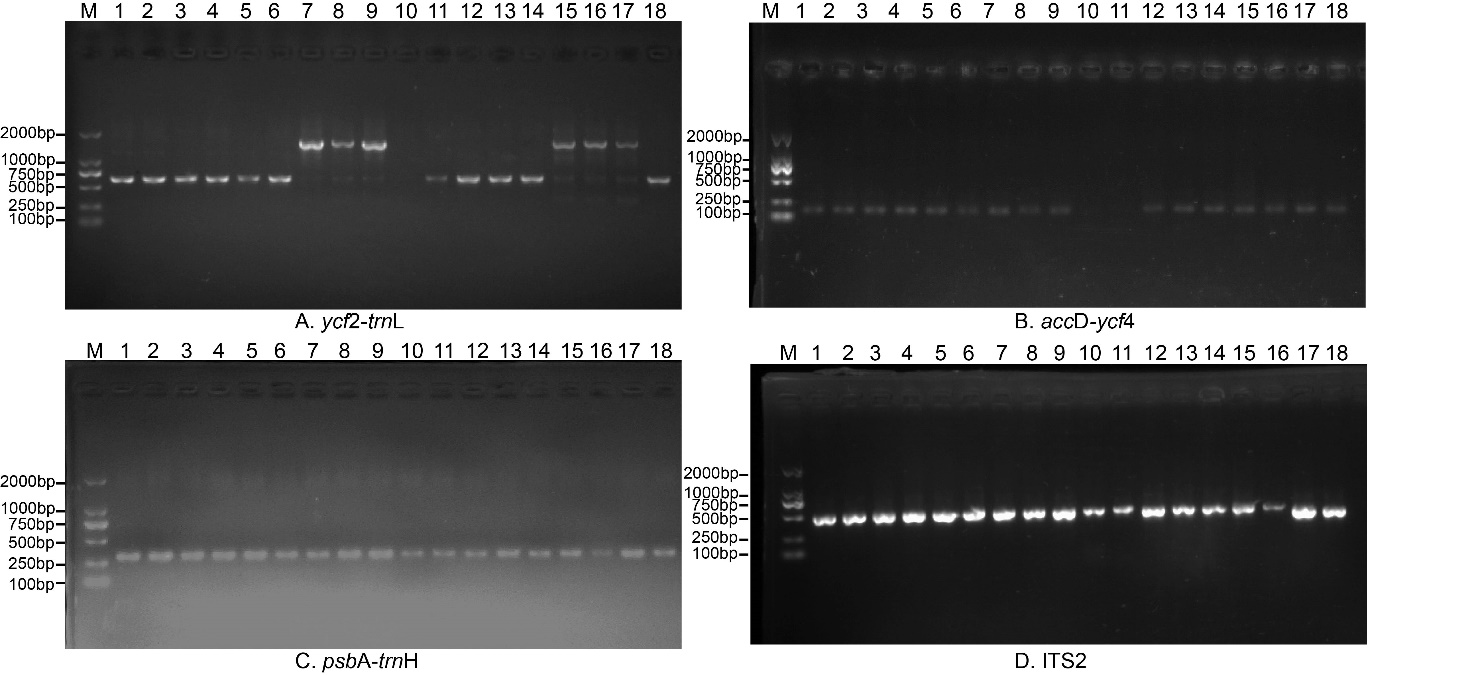


Figure S1. The PCR amplification results of (A) *ycf*2-*trn*l, (B) *acc*D-*ycf*4, (C) *psb*A-*trn*H, (D) ITS2. M: Maker DL 2000; CK: Control Check; Sample order: 1-3: *Ligusticum sinense* (TCMCQ1-3), 4-6: *Ligusticum jeholense* (TCMLN1-3), 7-9: *Conioselinum vaginatum* (TCMXJ1-3), 10: Ligusticum sp. (TCMSC1), 11: *Ligusticum sinense* (2017072501), 12-14: *Ligusticum jeholense* (2017090101, 2021LGB01, 2021LGB02), 15-17: *Conioselinum vaginatum* (2018080504, 2018080506, 2017072522), 18. *Ligusticum jeholense* (2017091301).
